# The TGF-β System As a Potential Pathogenic Player in Disease Modulation of Amyotrophic Lateral Sclerosis

**DOI:** 10.3389/fneur.2017.00669

**Published:** 2017-12-15

**Authors:** Sebastian Peters, Eva Zitzelsperger, Sabrina Kuespert, Sabine Iberl, Rosmarie Heydn, Siw Johannesen, Susanne Petri, Ludwig Aigner, Dietmar R. Thal, Andreas Hermann, Jochen H. Weishaupt, Tim-Henrik Bruun, Ulrich Bogdahn

**Affiliations:** ^1^Department of Neurology, University Hospital Regensburg, Regensburg, Germany; ^2^Department of Hematology, University Hospital Regensburg, Regensburg, Germany; ^3^Department of Neurology, University Hospital MHH, Hannover, Germany; ^4^Institute of Molecular Regenerative Medicine, Spinal Cord Injury and Tissue Regeneration Center Salzburg, Paracelsus Medical University, Salzburg, Austria; ^5^Department for Neuroscience, Laboratory for Neuropathology, University of Leuven, Leuven, Belgium; ^6^Department of Neurology, Technische Universität Dresden and German Center for Neurodegenerative Diseases (DZNE), Research Site Dresden, Dresden, Germany; ^7^Department of Neurology, University Hospital Ulm, Ulm, Germany

**Keywords:** amyotrophic lateral sclerosis, TGF-β, immunity, adult neurogenic niche, fibrosis

## Abstract

Amyotrophic lateral sclerosis (ALS) represents a fatal orphan disease with high unmet medical need, and a life time risk of approx. 1/400 persons per population. Based on increasing knowledge on pathophysiology including genetic and molecular changes, epigenetics, and immune dysfunction, inflammatory as well as fibrotic processes may contribute to the heterogeneity and dynamics of ALS. Animal and human studies indicate dysregulations of the TGF-β system as a common feature of neurodegenerative disorders in general and ALS in particular. The TGF-β system is involved in different essential developmental and physiological processes and regulates immunity and fibrosis, both affecting neurogenesis and neurodegeneration. Therefore, it has emerged as a potential therapeutic target for ALS: a persistent altered TGF-β system might promote disease progression by inducing an imbalance of neurogenesis and neurodegeneration. The current study assessed the activation state of the TGF-β system within the periphery/in life disease stage (serum samples) and a late stage of disease (central nervous system tissue samples), and a potential influence upon neuronal stem cell (NSC) activity, immune activation, and fibrosis. An upregulated TGF-β system was suggested with significantly increased TGF-β1 protein serum levels, enhanced TGF-β2 mRNA and protein levels, and a strong trend toward an increased TGF-β1 protein expression within the spinal cord (SC). Stem cell activity appeared diminished, reflected by reduced mRNA expression of NSC markers Musashi-1 and Nestin within SC—paralleled by enhanced protein contents of Musashi-1. Doublecortin mRNA and protein expression was reduced, suggesting an arrested neurogenesis at late stage ALS. Chemokine/cytokine analyses suggest a shift from a neuroprotective toward a more neurotoxic immune response: anti-inflammatory chemokines/cytokines were unchanged or reduced, expression of proinflammatory chemokines/cytokines were enhanced in ALS sera and SC postmortem tissue. Finally, we observed upregulated mRNA and protein expression for fibronectin in motor cortex of ALS patients which might suggest increased fibrotic changes. These data suggest that there is an upregulated TGF-β system in specific tissues in ALS that might lead to a “neurotoxic” immune response, promoting disease progression and neurodegeneration. The TGF-β system therefore may represent a promising target in treatment of ALS patients.

## Introduction

Amyotrophic lateral sclerosis (ALS) is a fatal neurodegenerative disease with a progressive loss of upper and lower motor neurons (1). The broad clinical phenotype in combination with few disease biomarkers results in late diagnosis with advanced disease progression (2, 3). In this context, the TGF-β system has emerged as a potential target due to its involvement in essential cellular and physiological processes, such as proliferation, cell differentiation, and growth; further, TGF-β is involved in immune regulation, stem cell activity and fibrosis. Effects are strictly context and dose dependent. Consequently, a persistent dysregulation may lead to a disturbed homeostasis on several levels—finally resulting in an imbalance of degenerative and regenerative processes (4). Confirming former studies showing enhanced circulating TGF-β1 levels within serum, plasma, and CSF of ALS patients (5, 6), recent studies demonstrated astrocyte-derived TGF-β1, upregulated in the spinal cord (SC) of symptomatic mSOD1 mice and sporadic ALS patients, to be a detrimental factor correlating with disease progression (7). Human skeletal muscle biopsies and skeletal muscle samples from mSOD1 mice revealed enhanced TGF-β1 mRNA and protein expression with increased signaling, fibrosis, and disease progression (8, 9). In contrast to the acute anti-inflammatory properties of TGF-β, a persistent elevated system activity might promote ALS progression by interacting with three different systems, namely (i) the immune response, (ii) the activity of the adult neurogenic niche, and (iii) fibrotic scarring.

A growing number of *in vitro* and *in vivo* experiments have indicated immune and inflammatory abnormalities to contribute to the pathogenesis and progression of ALS (10). Neuronal injury occurs on sites of inflammation populated by resident and infiltrating immune cells, which act in concert with secreted inflammatory modulators and expressed surface receptors of activated microglial cells (11). Inflammation-mediated loss of neurons is common for neurodegenerative disorders including ALS (11). Activated microglia and reactive astrocytes releasing cytokines and chemokines as their main effector molecules have been identified as key components of neuronal loss within the central nervous system (CNS) (12). Basically, ALS disease course may be sectioned into two major immunological phases: a presymptomatic or stable phase with a predominantly pronounced anti-inflammatory T2 immune response and a progressive phase with a distinct tendency toward a proinflammatory T1 immunity (13). This successive process of immune polarization was shown to occur within several months in murine ALS models, whereas several years for the human situation (10, 13, 14).

In addition to neuronal loss, inflammation also reduces neurogenesis in many neurodegenerative disorders and age-associated brain pathologies such as dementia (15–20). However, recent studies have refuted the dogma that the adult CNS is unable to self-repair or regenerate (21, 22). Main mammalian regions of neurogenesis are the subgranular zone (SGZ) of the dentate gyrus, the subventricular zone (SVZ) of the lateral ventricles, and the olfactory bulb (23, 24). In addition, different *in vivo* studies have demonstrated further loci of neurogenesis in the CNS (25–31)—in relation to ALS, the SC of mice (32), rats (33), and primates (34). Inflammation influences neurogenesis *via* affecting proliferation, cell survival and death, but also the integration of newly generated neurons into the established neuronal network and finally the molecular characteristics of the assimilated and surviving neurons (35). Again, main effectors are activated microglia cells with proinflammatory cytokines/chemokines, whose expression levels correlate with the decrease in hippocampal neurogenesis (36, 37). With respect to stem cell activity and adult neurogenesis, constantly elevated TGF-β levels have been shown to reduce the activity of human hematopoietic (38) and neuronal stem cells within rats (39). Thus, a reduced activity of the adult neurogenic niche, induced by an enhanced proinflammatory milieu on the one hand, and by persistently enhanced TGF-β levels on the other, impairs the compensatory mechanisms for enhanced neuronal loss, leading to an aggravation of the disease course.

Fibrosis, a common response to tissue damage and chronic inflammation, impedes repair processes and the replacement of functional tissue by the exaggerated deposition of extracellular matrix (ECM) components. As a result, functional tissue is replaced by indestructible scars (40). Enhanced fibrotic processes within the CNS are described following stroke and for neurodegenerative disorders (41) and might therefore promote the progression of ALS indirectly by replacing areas of neuronal loss with excessive scar tissue. This hypothesis is strengthened by a recent murine *in vivo* study, showing enhanced muscle fibrosis which is induced by an enhanced TGF-β signaling in symptomatic hSOD1^G93A^ mice (9). For the situation within the CNS, astrocytes and microglia, surrounding the inflammatory center of lesions, are main cellular sources of increased TGF-β1 in CNS disorders (42). TGF-β1 as well as IL-1β act directly on fibroblasts or their precursors to induce a profibrotic phenotype (43). Cell culture experiments have demonstrated that pericytes also express TGF-β receptor 1 (TGF-βRI) and TGF-β receptor 2 (TGF-βRII). Upon stimulation, these cells induce the production of ECM and the expression of α-smooth muscle actin (44, 45). In addition, TGF-β1 has antiapoptotic properties on myofibroblasts (46) and attracts fibroblasts *in vitro* (47).

This human study pursues the goal to further clarify the role of TGF-β within the periphery (serum samples) and at the final state of ALS (postmortem CNS tissue), regarding the context of immune activation, stem cell activity and fibrosis.

## Materials and Methods

### Human Serum

Human serum samples were taken from female and male ALS patients (*n* = 37) and female and male healthy controls (*n* = 11) at the University Hospital of Regensburg (Table 1). Sample collection was performed to evaluate peripheral circulating levels of TGF-β and parameters of the immune system. Sera were obtained according to the regulations of the ethics committee at the University of Regensburg (ethics approval: 15-101-0106). All patients were regularly seen in the ALS outpatient’s clinic, for all measures taken patients were given oral and written information—written informed consent was then obtained in each case. The principles of the declaration of Helsinki for all human clinical scientific work were strictly observed (according to the latest revision 2013, Fortaleza, Brazil).

**Table 1:** Characteristics of controls and ALS patients for the analysis of sera with the individual ages on the date of measurement (healthy volunteers) and the ages at diagnosis as well as sample donation (ALS patients).

		Patient no.	Age at diagnosis	Age at sample donation	Sex	Diagnosis
Serum samples	Controls	1	–	38	Male	Healthy volunteer
		2	–	29	Female	Healthy volunteer
		3	–	29	Female	Healthy volunteer
		4	–	52	Female	Healthy volunteer
		5	–	56	Female	Healthy volunteer
		6	–	32	Male	Healthy volunteer
		7	–	40	Male	Healthy volunteer
		8	–	37	Female	Healthy volunteer
		9	–	42	Male	Healthy volunteer
		10	–	29	Female	Healthy volunteer
		11	–	39	Female	Healthy volunteer

	ALS patients	1	48	50	Female	ALS
		2	40	42	Male	ALS
		3	75	77	Male	ALS
		4	68	68	Female	ALS
		5	65	67	Male	ALS
		6	25	26	Male	ALS
		7	48	50	Female	ALS
		8	72	73	Male	ALS
		9	49	50	Male	ALS
		10	54	56	Male	ALS
		11	41	41	Male	ALS
		12	35	35	Female	ALS
		13	48	48	Female	ALS
		14	43	43	Male	ALS
		15	65	65	Female	ALS
		16	51	51	Female	ALS
		17	54	55	Male	ALS
		18	60	61	Female	ALS
		19	58	58	Male	ALS
		20	45	46	Male	ALS
		21	50	50	Male	ALS
		22	27	27	Male	ALS
		23	45	45	Male	ALS
		24	55	55	Male	ALS
		25	61	61	Male	ALS
		26	60	60	Male	ALS
		27	65	65	Female	ALS
		28	42	43	Female	ALS
		29	60	60	Male	ALS
		30	44	45	Female	ALS
		31	46	47	Male	ALS
		32	50	50	Male	ALS
		33	38	39	Male	ALS
		34	55	56	Male	ALS
		35	59	59	Male	ALS
		36	68	69	Male	ALS
		37	34	35	Male	ALS

### Human Tissue

Human postmortem cryopreserved and paraffin-embedded tissue from the SC, the motor cortex (MC), and the occipital lobe (OL) as an internal control, were obtained from the German Motor Neuron Disease Network (MND-Network, Albert Ludolph, Ulm), from female and male ALS patients (*n* = 18) and female and male healthy controls (*n* = 17) and was kindly provided by Prof. Dr. Dietmar Thal and Dr. Susanne Petri (Table 2). We included the MC in the analysis of the current study since the characteristic of ALS is the degeneration of the upper and lower motor neurons. The analysis of SC lysates covered the investigation of the surrounding milieu of the lower motor neurons since they originate within the SC and project to the muscles. For the upper motor neurons, their origin is within the MC or the brain stem. Therefore, we investigated whether these systems are involved or might promote ALS disease progression, we included the MC for the upper motor neuron. For the OL, it is known that this area is relatively unaffected by ALS and therefore not involved in the immediate diseases progression. Due to this, we included this area in the analysis as an internal control, to survey if alterations seen in the SC and MC are specific. Human tissue was obtained according to different ethical votes from the ethics committee at the University of Regensburg (ethics approval: 15-101-0053), the MND-network votes from the ethics committee at the University of Ulm (ethics approval: 19/12-2012), and the ethics committee at the University of Regensburg (ethics approval: 13-103-0056). Autopsies were explicitly part of the written informed consent for all patients, being seen in the context of the German Motor Neuron Network (funded by the BMBF).

**Table 2:** Characteristics of controls and ALS patients for the analysis of post mortem tissue samples with the individual ages of the controls and ALS patients at disease onset and day of death.

		Patient no.	Age at onset	Age at death	Sex	Diagnosis	Spinal cord	Motor cortex	Occipital lobe
Postmortem CNS tissue	Controls	1	–	43	Male	Non-ALS	Yes	Yes	Yes
		2	–	61	Male	Non-ALS	Yes	Yes	Yes
		3	–	66	Male	Non-ALS	Yes	Yes	Yes
		4	–	64	Male	Non-ALS	Yes	Yes	Yes
		5	–	35	Male	Non-ALS	Yes	Yes	Yes
		6	–	59	Male	Non-ALS	Yes	Yes	Yes
		7	–	67	Female	Non-ALS	Yes	Yes	Yes
		8	–	75	Female	Non-ALS	Yes	Yes	Yes
		9	–	74	Male	Non-ALS	Yes	Yes	Yes
		10	–	53	Female	Non-ALS	Yes	Yes	Yes
		11	–	44	Male	Non-ALS	Yes	No	No
		12	–	71	Female	Non-ALS	Yes	No	No
		13	–	62	Male	Non-ALS	Yes	No	No
		14	–	84	Female	Non-ALS	Yes	No	No
		15	–	70	Male	Non-ALS	Yes	No	No
		16	–	78	Female	Non-ALS	No	Yes	No
		17	–	42	Female	Non-ALS	No	Yes	No

	ALS patients	1	58	61	Male	ALS	Yes	Yes	No
		2	74	75	Male	ALS	No	Yes	No
		3	65	66	Female	ALS	Yes	Yes	Yes
		4	59	61	Male	ALS	Yes	Yes	Yes
		5	68	72	Female	ALS	No	Yes	Yes
		6	n.a.	75	Male	ALS	Yes	Yes	Yes
		7	73	74	Female	ALS	Yes	Yes	No
		8	n.a.	57	Male	ALS	Yes	Yes	Yes
		9	39	43	Male	ALS	No	Yes	Yes
		10	60	61	Female	ALS	Yes	Yes	Yes
		11	52	54	Female	ALS	Yes	Yes	No
		12	74	75	Male	ALS	Yes	Yes	No
		13	58	60	Female	ALS	Yes	Yes	No
		14	54	57	Male	ALS	Yes	Yes	No
		15	66	70	Male	ALS	Yes	Yes	No
		16	72	74	Female	ALS	Yes	Yes	No
		17	65	68	Male	ALS	Yes	Yes	No
		18	70	71	Female	ALS	Yes	Yes	No

### Determination of CD34^+^ Cells

Human CD34^+^ positive cells were determined as previously described (48). Briefly, 1 ml of donor blood was lysed in 9 ml of NH_4_CL lysis buffer for 5 min. Cells were then washed twice in phosphate-buffered saline (PBS, Sigma– Aldrich, St. Louis, MO, USA) containing 2% fetal bovine serum (FBS, PAA Laboratories, Pasching, Austria). For flow cytometric analysis cells were stained for 30 min at 4°C with combinations of the following murine monoclonal antibodies: anti-CD45-fluorescein-isothiocyanate (FITC, clone HI30; BD Pharmingen, Franklin Lakes, NJ, USA) and CD34-allophycocyanin (APC, clone 581, Biolegend, San Diego, CA, USA). After the labeling procedure, cells were washed twice with PBS2%FBS and subsequently analyzed using a Becton Dickinson FACSCalibur^®^ flow cytometer. The threshold was defined as less than 2% of cells stained with the respective isotype antibody being positive. Approximately 3 × 10^5^–5 × 10^5^ events were analyzed to ensure statistical validity.

### Quantitative Real-Time Polymerase Chain Reaction (qRT-PCR)

For mRNA analysis, 30 mg of the respective tissue was taken for RNA isolation using the DNA/RNA/miRNA Universal Kit (Qiagen, Hilden, Germany). Following determination of RNA content (100 ng RNA per 20 µl), the RNA was reversely transcribed into first strand cDNA with iScript cDNA Synthesis Kit (BioRad, Hercules, CA, USA) according to manufacturer’s recommendations. For mRNA analysis, qRT-PCR was performed using a CFX96 Touch Real Time PCR Detection System (BioRad, Hercules, CA, USA). All primer pairs [TGF-β1 (qHsaCID0017026), TGF-β2 (qHsaCID0018360), TGF-βRI (qHsaCID0009475), TGF-βRII (qHsaCID0016240), Nestin (qHsaCED0044457), SOX-2 (qHsaCED0036871), MSI1 (qHsaCID0008192), DCX (qHsaCID0010869), fibronectin (qHsaCID0012349), and collagen IV (qHsaCID0010223)] were ready-to-use standardized and were mixed with the respective ready-to-use Mastermix solution (Sso Advanced Universial SYBR Green Supermix, BioRad, Hercules, CA, USA) according to manufacturer’s instructions (BioRad Prime PCR Quick Guide). As template, 0.25 µl of respective cDNA was used. H_2_O was used as a negative control for qRT-PCR. For relative quantification, housekeeping gene GNB2L1 was used. Afterward, BioRad CFX Manager 3.1 was used to quantify mRNA-level relative to GNB2L1 mRNA.

### Western Blotting

For protein analysis, about 30 mg of the respective tissue was lysed using T-PER^®^ Tissue Protein Extraction Reagent (Thermo Scientific, Braunschweig, Germany) according manufactory instructions. Afterwards, protein concentrations were determined using Pierce Coomassie Plus Assay Kit (Life Technologies). SDS-acrylamid-gels (12%) were produced using TGX Stain Free™ Fast Cast™ Acrylamid Kit (BioRad, Hercules, CA, USA) according to manufactory instructions. Protein samples (20 µl) were diluted 1:4 with Lämmli-buffer (6.5 µl, Roti^®^-Load1, Roth, Karlsruhe, Germany), incubated at 60°C for 30 min and loaded on the gel with the entire volume of the protein solution. Separation of proteins was performed by electrophoresis using Power Pac Basic Power Supply (BioRad, Hercules, CA, USA) and Mini Protean Tetra cell electrophoresis chamber (BioRad, Hercules, CA, USA) (200 V, 45 min). Following electrophoresis, the proteins were blotted using Trans-Blot Turbo Transfer System (BioRad, Hercules, CA, USA). All materials for western blotting were included in Trans Blot Turbo RTA PVDF-Midi Kit (BioRad, Hercules, CA, USA). The PVDF-membrane for blotting procedures was activated in methanol (Merck Darmstadt, Germany) and equilibrated in 1× transferbuffer. Following blotting (25 V, 1 A, 30 min), membranes were washed (3×, 10 min, RT) with 1× TBS (Roth, Karlsruhe, Germany) containing 0.05% Tween-20 (Roth, Karlsruhe, Germany). Afterward, the membranes were blocked with 5% BSA (Albumin-IgG-free, Roth, Karlsruhe, Germany), diluted with TBS-T for 1 h at RT, the primary antibodies (diluted in 0.5% BSA in TBS-T) were added and incubated at 4°C for 2 days [rabbit anti-TGF-β1 (1:300; Acris), rabbit anti-TGF-β2 (1:500; BioVision), rabbit anti-TGF-βRI (1:1,000; Abcam), rabbit anti-TGF-βRII (1:1,000; Aviva), rabbit anti-Nestin (1:100; Abcam), rabbit anti-SOX-2 (1:750; Millipore), rabbit anti-MSI1 (1:2,000; Abcam), rabbit anti-DCX (1:1,000; Cellsignaling), rabbit anti-Fibronectin (1:250; ProteinTech), and rabbit anti-CollagenIV (1:1,000; Abcam)]. Next, membranes were washed in TBS-T (3 × 10 min, RT) and incubated with the secondary antibody (1 h, RT). Following incubation, blots were washed with TBS-T, emerged using Luminata™Forte Western HRP Substrate (Millipore, Millipore, Germany) and bands were detected with a luminescent image analyzer (ImageQuant LAS 4000, GE Healthcare, Munich, Germany). Afterward, the blots were washed in TBS-T (3 × 10 min, RT) and blocked with 5% BSA diluted in TBS-T (1 h, RT). For housekeeper comparison, the membranes were incubated with HRP-conjugated anti GAPDH (1:2,000 in 0.5% BSA, 4°C, over night; Cell Signaling). On the following day, blots were emerged using Luminata™Forte Western HRP Substrate (Millipore, Darmstadt, Germany) and bands were detected with a luminescent image analyzer (ImageQuant LAS 4000, GE Healthcare, Munich, Germany). Finally, the blots were washed with TBS-T (3×, 5 min) and stained using 1× Roti Blue solution (Roth, Karlsruhe, Germany) and dried at RT. Blots were analyzed using Image Studio Lite Software (Licor, NE, USA). Due to the limitation of available tissue/material we had to focus on some parts for our analysis. Therefore, we think that assessing the active form of TGF-β on the protein level is sufficient for the current study, since only the active form binds to the receptor and activates/mediates its intracellular effects. Whole blots for the representative ones within the manuscript are shown in Figures S1–S9 in Supplementary Material.

### Electrochemoluminescence

To analyse TGF-β1 and 2 ligand as well as cytokine expression, about 30 mg of CNS tissue were lysed using T-PER^®^ Tissue Protein Extraction Reagent (Thermo Scientific, Braunschweig, Germany) according manufactory instructions. Afterward, protein concentration was determined using Pierce Coomassie Plus Assay Kit (Life Technologies) and the final concentration was adjusted to 1 µg/µl. For electrochemoluminescence (Mesoscale Discovery, MD, USA) 25 µl of the serum samples as well as protein supernatants were used. Serum concentrations/tissue expression of TGF-β ligands and cytokines/chemokines were measured using customized human TGF-β U-Plex assay and V-Plex Human Biomarker 40-Plex Kit. The assay procedures were performed according to manufacturer’s instructions.

### Statistics

For graph design and statistical comparisons, GraphPad Prism 6 was employed. All parameters were tested for Gaussian distribution using D’Augostino-Pearson omnibus normality test. Afterward, all parameters were analyzed using a two-tailed Student’s *t*-test or Mann–Whitney test, depending on Gaussian distribution. Data are presented as mean ± SEM for data with normal distribution and as median with interquartile range for data with no normal distribution. Significance was taken at *p* ≤ 0.05.

## Results

### ALS Patients Exhibit Enhanced Circulating TGF-β1 Serum Levels

**Figure 1:**
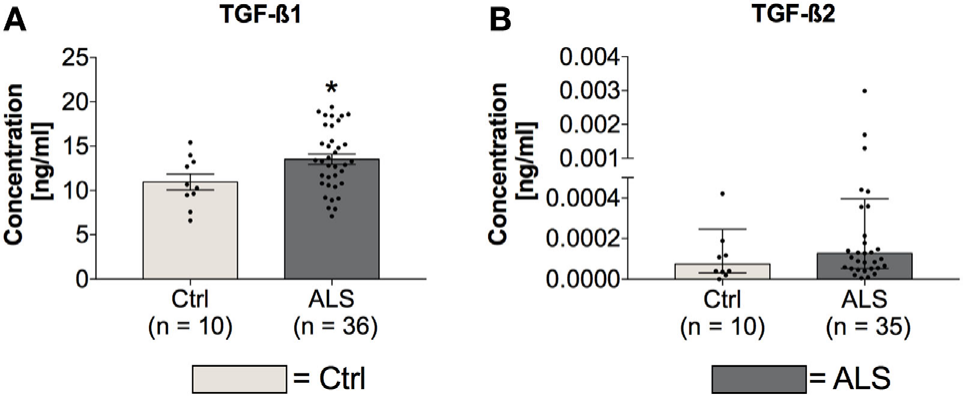
Serum concentrations of TGF-β1 and TGF-β2. Amyotrophic lateral sclerosis (ALS) patients exhibited enhanced circulating TGF-β1 serum levels **(A)**, whereas TGF-β2 amounts were unchanged **(B)**. *p < 0.05 vs. Controls (Ctrl). Numbers of patients are given in brackets; all parameters were tested for Gaussian distribution using D’Augostino-Pearson omnibus normality test. Two-tailed unpaired Student’s *t*-test for TGF-β1, data represent mean ± SEM; two-tailed Mann–Whitney test for TGF-β2, data represent median with interquartile range.

In order to investigate whether circulating TGF-β ligand levels differ between controls and ALS patients, serum levels of TGF-β1 and TGF-β2 were determined *via* electrochemiluminescence. Statistical analysis revealed significantly enhanced circulating serum levels of TGF-β1 in ALS patients versus healthy controls (*p* = 0.040; Figure 1A); TGF-β2 level increases were not significant (*p* = 0.224; Figure 1B). These results demonstrate an activated peripheral TGF-β system within ALS patients.

### ALS Patients Exhibit an Enhanced Peripheral Proinflammatory Immune Profile

**Figure 2:**
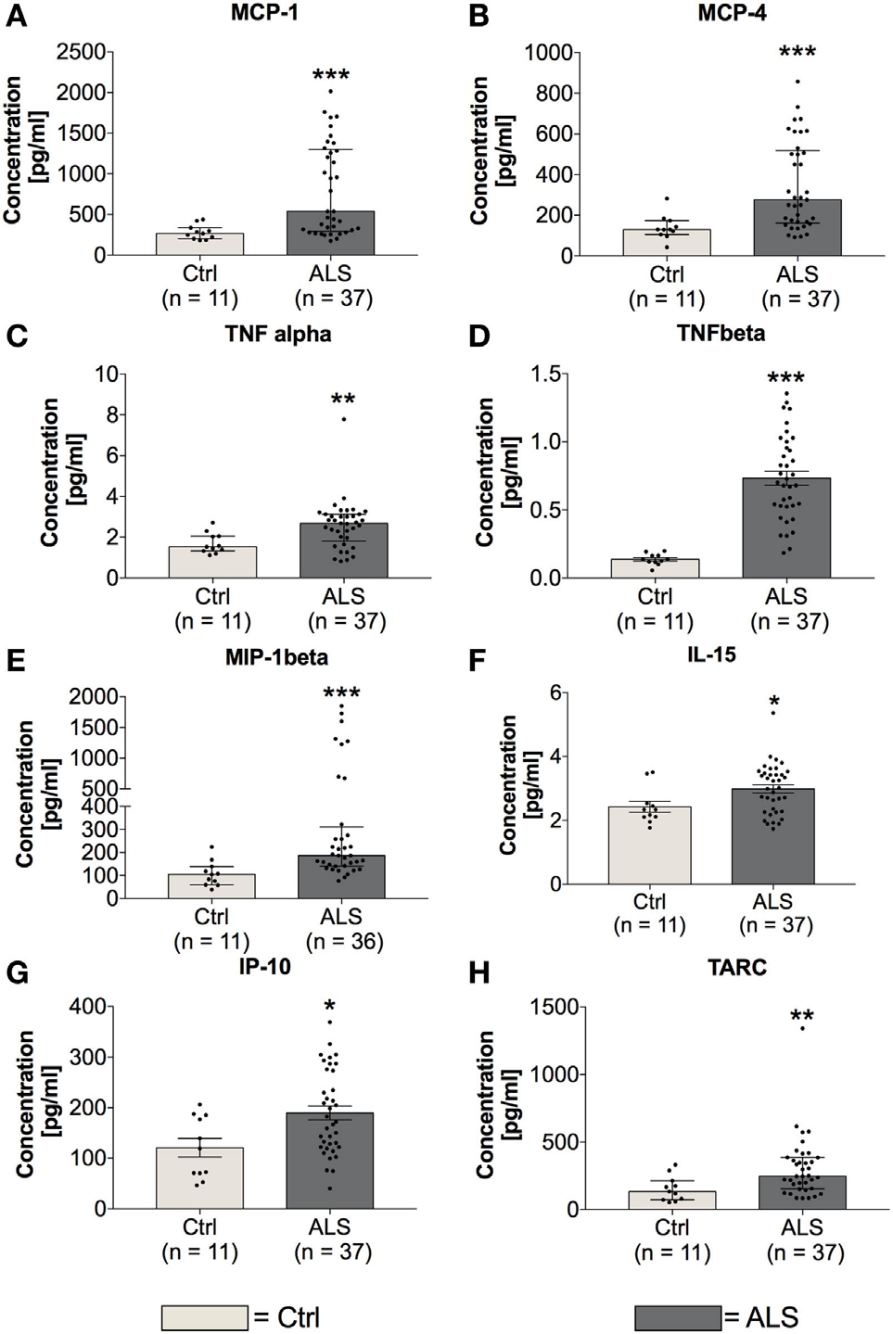
Serum concentrations of proinflammatory cytokines and chemokines. Amyotrophic lateral sclerosis (ALS) patients exhibited an upregulated peripheral proinflammatory immune response **(A–H)**. Circulating levels of the proinflammatory chemokines MCP-1 **(A)** and MCP-4 **(B)** as well as proinflammatory cytokines TNFα **(C)**, TNFβ **(D)**, MIP-1β **(E)**, IL-15 **(F)**, IP-10 **(G)**, and TARC **(H)** were significantly enhanced in ALS patients compared to healthy controls (Ctrl). *p < 0.05 vs. Ctrl, **p < 0.01 vs. Ctrl, ***p < 0.001 vs. Ctrl. Numbers of patients are given in brackets; All parameters were tested for Gaussian distribution using D’Augostino-Pearson omnibus normality test. Two-tailed Mann–Whitney test for TNFα, MCP-1, MCP-4, MIP-1β, IL-15, and TARC, data represent median with interquartile range; two-tailed unpaired Student’s *t*-test for TNFβ and IP-10, data represent mean ± SEM.

**Figure 3:**
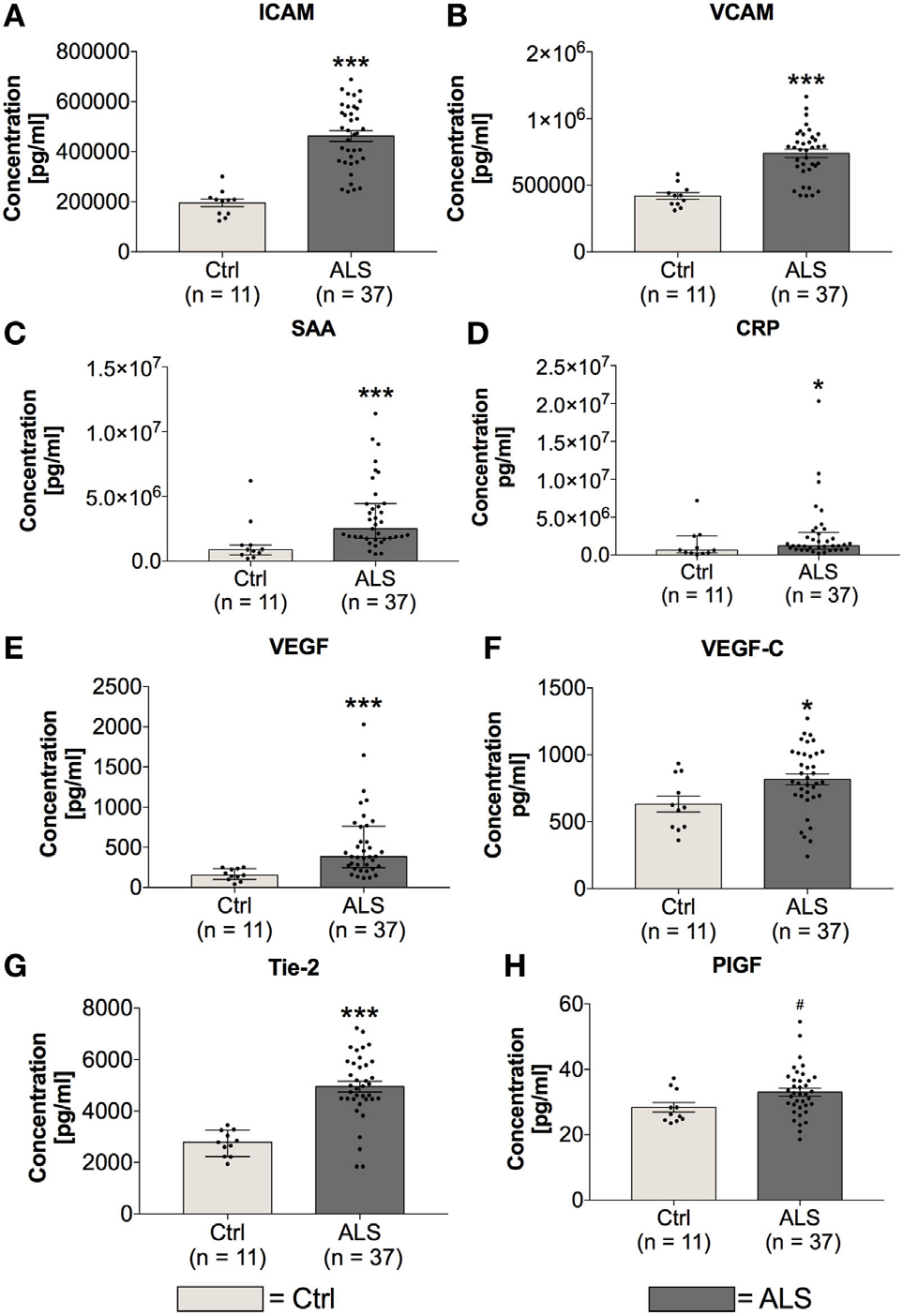
Serum concentrations of vascular and general inflammatory markers. Amyotrophic lateral sclerosis (ALS) patients showed a significantly enhanced expression profile of vascular and endothelial factors ICAM **(A)** and VCAM **(B)**, as well as general inflammatory markers SAA **(C)** and CRP **(D)**. Further, circulating levels of angiogenic factors including VEGF **(E)**, VEGF-C **(F)**, Tie-2 **(G)**, and PIGF **(H)** were increased in sera of ALS patients. *p < 0.05 vs. controls (Ctrl), ***p < 0.001 vs. Ctrl; # = trend, *p* = 0.0593. Numbers of patients are given in brackets. All parameters were tested for Gaussian distribution using D’Augostino-Pearson omnibus normality test. Two-tailed Mann–Whitney test for SAA, CRP, and VEGF, data represent median with interquartile range; two-tailed unpaired Student’s *t*-test for ICAM, VCAM, VEGF-C, Tie-2, and PIGF, data represent mean ± SEM.

In order to investigate the activation state of the peripheral immune response of controls and ALS patients, expression levels of cytokines, chemokines, vascular, and angiogenic factors were determined *via* electrochemiluminescence. Statistical analysis indicated significantly enhanced concentrations of MCP-1 (*p* = 0.001; Figure 2A) and MCP-4 (*p* = 0.001; Figure 2B) in serum of ALS patients compared to healthy controls. Further, proinflammatory cytokines TNFα (*p* = 0.006; Figure 2C) and TNFβ (*p* < 0.0001; Figure 2D) were found to be increased in ALS sera. In addition, concentrations of MIP-1β (*p* = 0.0002; Figure 2E), IL-15 (*p* = 0.033; Figure 2F), IP-10 (*p* = 0.014; Figure 2G), and TARC (*p* = 0.004; Figure 2H) were significantly enhanced in ALS patients compared to healthy controls. In addition, ALS patients displayed an increased amount of vascular factors ICAM (*p* < 0.001; Figure 3A) and VCAM (*p* < 0.001; Figure 3B). Finally, ALS patients exhibited significantly enhanced circulating levels of the general proinflammatory markers SAA (*p* = 0.0006; Figure 3C) and CRP (*p* = 0.022; Figure 3D) as well as angiogenic factors VEGF (*p* < 0.0001; Figure 3E), VEGF-C (*p* = 0.027; Figure 3F), Tie-2 (*p* < 0.001; Figure 3G), and a strong trend toward increased levels of PIGF (*p* = 0.059; Figure 3H). These results demonstrate a more pronounced proinflammatory immune response in ALS patients compared to controls.

### Activity of CD34^+^ Hematopoietic Stem Cells Seems to be Inhibited in ALS Patients

**Figure 4:**
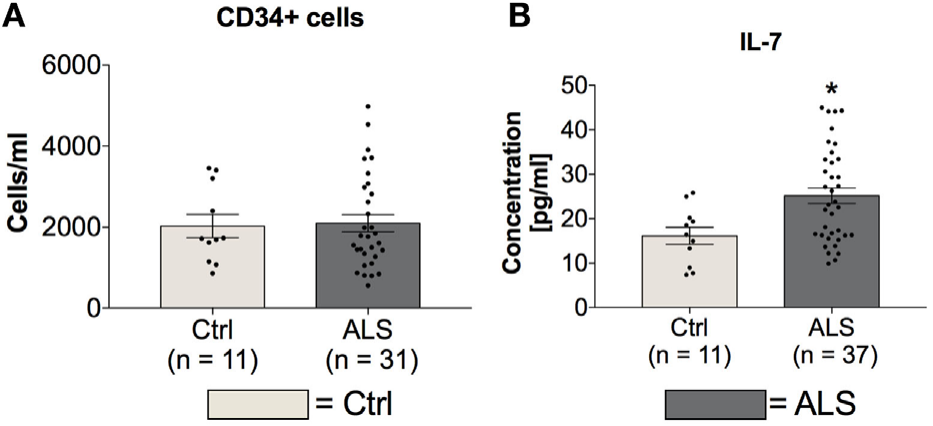
Blood concentrations of CD34^+^ cells and serum concentrations IL-7. Sera of Amyotrophic lateral sclerosis (ALS) patients displayed unchanged levels of CD34^+^ positive hematopoietic stem cells **(A)** and cell differentiation stimulating factor IL-7 **(B)**. *p < 0.05 vs. controls (Ctrl). Numbers of patients are given in brackets; all parameters were tested for Gaussian distribution using D’Augostino-Pearson omnibus normality test. Two-tailed unpaired Student’s *t*-test for CD34^+^ cells and IL-7, data represent mean ± SEM.

In order to investigate possible differences in amount and activity of hematopoietic stem cells of controls and ALS patients, serum concentrations of CD34^+^hematopoietic stem cells and IL-7 as a stem cell proliferation/differentiation promoting factor were determined. Statistical analysis revealed unchanged serum levels of CD34^+^ hematopoietic stem cells whereas the concentration of IL-7 was significantly increased in sera of ALS patients (*p* = 0.012; Figure 4B). These results might reflect an inhibited activity of CD34^+^ cells.

### ALS Patients Exhibit an Upregulated Activity of the TGF-β System at Final Disease Stage in Postmortem Tissue

**Figure 5:**
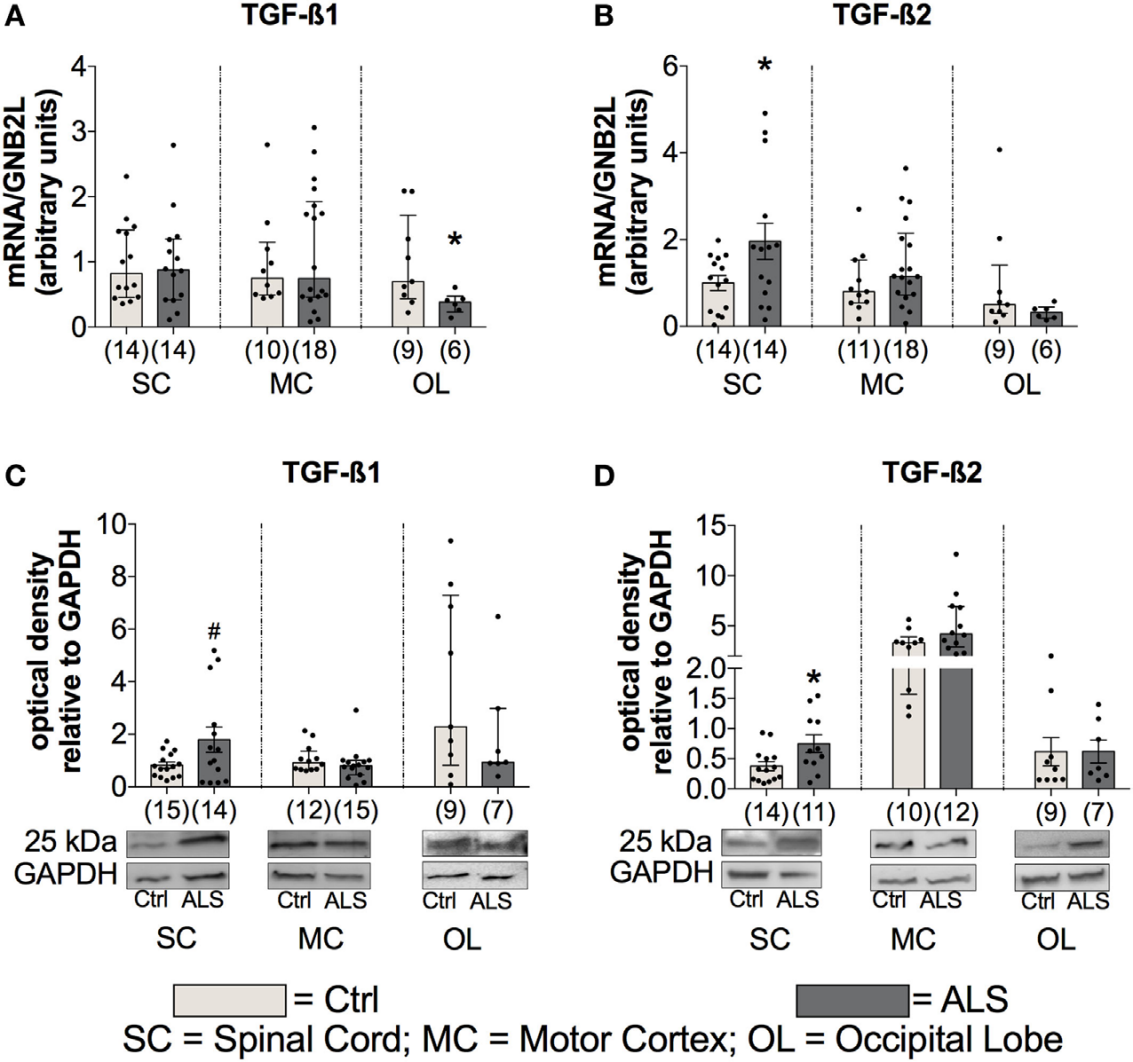
Tissue concentrations of TGF-β1 and TGF-β2. Tissue of Amyotrophic lateral sclerosis (ALS) patients displayed no alterations of TGF-β1 mRNA and protein expression levels **(A)**, but increased mRNA as well as protein expression levels of TGF-β2 **(B,D)** and a strong trend toward an enhanced protein expression of TGF-β1 within the spinal cord (SC; **D**). *p < 0.05 vs. controls (Ctrl), # = trend, *p* = 0.0538. Numbers of patients are given in brackets; all parameters were tested for Gaussian distribution using D’Augostino-Pearson omnibus normality test. Two-tailed unpaired Student’s *t*-test for TGF-β1 protein (SC) and TGF-β2 mRNA (SC) and protein (SC, OL), data represent mean ± SEM; two-tailed Mann–Whitney test for TGF-β1 mRNA (SC, motor cortex (MC)), occipital lobe (OL), protein (MC, OL), TGF-β2 mRNA (MC, OL), and TGF-β2 protein (MC), data represent median with interquartile range. TGF-β1 and TGF-β2 protein expression levels were quantified and represented relative to the expression levels of the housekeeping protein GAPDH.

In order to investigate whether the enhanced TGF-β ligand expression is also present at the final stage of the ALS disease course, postmortem SC, MC, and as an internal control, OL tissue was analyzed for TGF-β1 and TGF-β2 mRNA and protein levels, in controls and ALS patients. Statistical analysis revealed no differences in TGF-β1 mRNA expression within the SC (*p* = 0.769; Figure 5A), and MC (*p* = 0.944; Figure 5A) but a reduction within the OL of ALS patients compared to healthy controls (*p* = 0.036; Figure 5A). However, TGF-β2 mRNA expression patterns were increased in the SC (*p* = 0.043; Figure 5B) with no alteration in the MC (*p* = 0.220; Figure 5B) and OL (*p* = 0.259; Figure 5B). For the protein expression of the two ligands, there was a strong trend toward an enhanced protein expression of TGF-β1 (*p* = 0.054; Figure 5C) within the SC paralleled by a significantly increased expression of TGF-β2 (*p* = 0.023; Figure 5D). Within the MC and the OL, the TGF-β1 and -2 protein levels of ALS patients and healthy controls were comparable (TGF-β1(MC): *p* = 0.392; (OL): *p* = 0.299; Figure 5C/TGF-β2 (MC): *p* = 0.103; (OL): *p* = 0.993; Figure 5D).

**Figure 6:**
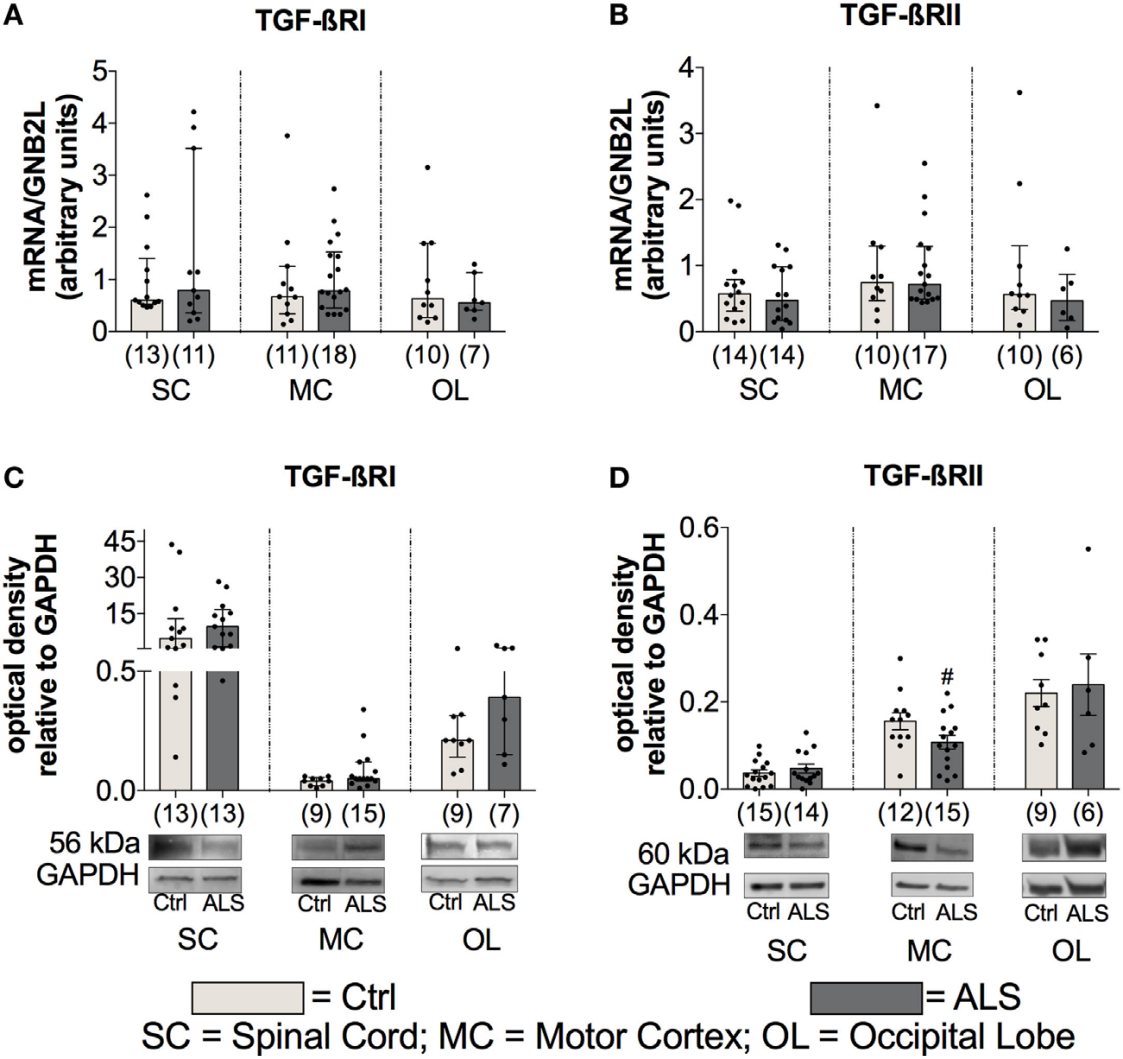
Tissue concentrations of TGF-β receptors. For the TGF-β receptors, there was no difference in the mRNA expression levels of the TGF-βRI **(A)** and the TGF-βRII **(B)**. For protein expression, there was no alteration for the the TGF-βRI **(C)** and only a trend toward a reduced protein expression of the TGF-βRII and only in motor cortex (MC; **D**) of Amyotrophic lateral sclerosis (ALS) patients compared to healthy controls (Ctrl). # = trend, *p* = 0.062. Numbers of patients are given in brackets; all parameters were tested for Gaussian distribution using D’Augostino-Pearson omnibus normality test. Two-tailed Mann–Whitney test for TGF-βRI, TGF-βRII mRNA expression levels and TGF-βRI protein expression levels, data represent median with interquartile range, two-tailed unpaired Student’s *t*-test for TGF-RII protein, data represent mean ± SEM. TGF-βRI and TGF-βRII protein expression levels were quantified and represented relative to the expression levels of the housekeeping protein GAPDH.

To further investigate the activation state of the TGF-β system, the mRNA as well as protein expression levels of the two receptors TGF-βRI and TGF-βRII were determined. Here, statistical analysis indicated no differences for mRNA expression levels for TGF-βRI or for TGF-βRII within the SC (TGF-βRI: *p* = 0.832; TGF-βRII: *p* = 0.626) the MC (TGF-βRI: *p* = 0.499; TGF-βRII: *p* = 0.951), and OL (TGF-βRI: *p* = 0.739; TGF-βRII: *p* = 0.476) (Figures 6A,B). For protein expression, no alterations for TGF-βRI were detectable at any of the three different areas (SC: *p* = 0.362; MC: *p* = 0.136; OL: *p* = 0.173; Figure 6C). Within the MC, there was a trend toward a reduced expression of the TGF-βRII (*p* = 0.062; Figure 6D) and the SC (*p* = 0.411) and the OL (*p* = 0.781) were unaffected.

These results indicate an enhanced TGF-β system activity implied by enhanced ligand expression levels and a tendency toward a local RII downregulation as a compensatory mechanism, which is explained by the autocrine loop regulation in the TGF-β system.

### ALS Patients Exhibit a Shift toward a Neurotoxic and Destructive Immune Profile at Final Disease Stage in Postmortem Tissue

**Figure 7:**
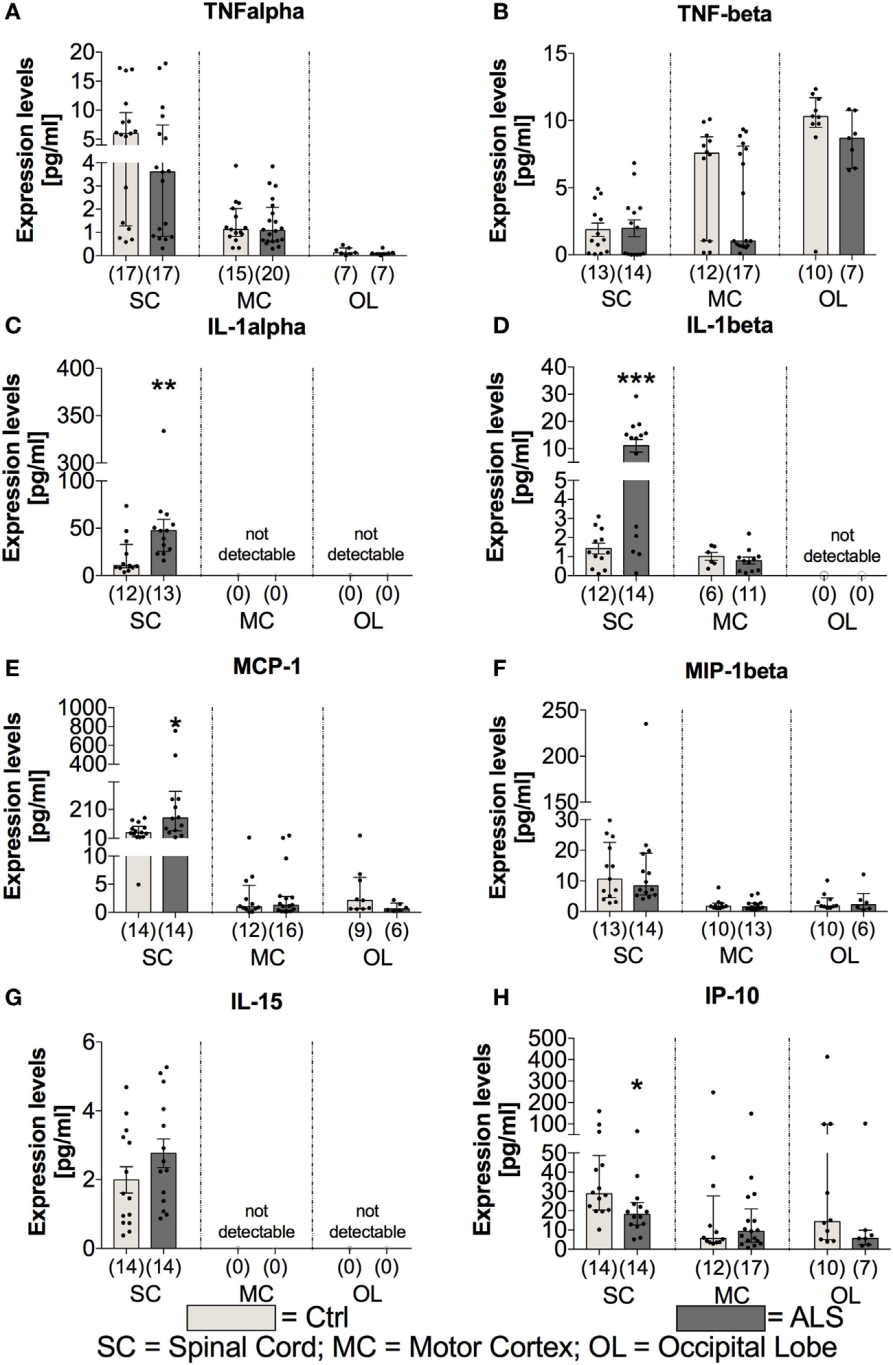
Tissue concentrations of proinflammatory cytokines and chemokines. Expression levels of proinflammatory cytokines where either unchanged [TNFα **(A)**, TNFβ **(B)**, significantly enhanced (IL-1α **(C)**, IL-1β **(D)**), IL-15 **(G)]**, or significantly reduced [IP-10, **(H)**] in amyotrophic lateral sclerosis (ALS) patients compared to healthy controls (Ctrl). The expression of the proinflammatory chemokine MCP-1 **(E)** was significantly increased, whereas the amounts of MIP-1β **(F)** were unaffected. Effects were only detectable within the spinal cord (SC). For the motor cortex (MC) and the occipital lobe (OL), the expression levels were unchanged within the two groups or not detectable. *p < 0.05 vs. Ctrl, **p < 0.01 vs. Ctrl, ***p < 0.001 vs. Ctrl. Numbers of patients are given in brackets; All parameters were tested for Gaussian distribution using D’Augostino-Pearson omnibus normality test. Two-tailed Mann–Whitney test for TNFα, TNFβ (MC, OL), IL-1α, MCP-1, MIP-1β, IP-10, data represent median with interquartile range; two-tailed unpaired Student’s *t*-test for TNFβ (SC), IL-1β, and IL-15, data represent mean ± SEM.

In order to investigate whether the proinflammatory milieu, seen in the periphery, is still present at the final disease stage and within the CNS tissue, postmortem SC, MC, and OL tissue was analyzed for the expression of cytokines, chemokines, vascular, and angiogenic factors. Statistical analysis revealed no differences for the proinflammatory cytokines TNFα (SC: *p* = 0.306; MC: *p* = 0.773; OL: *p* = 0.427) and TNFβ (SC: *p* = 0.896; MC: *p* = 0.283; OL: *p* = 0.161) in all three areas (Figures 7A,B). In contrast, IL-1α as well as IL-1β levels were significantly enhanced in the SC of ALS patients (IL-1α: *p* = 0.003; Figure 7C; IL-1β: *p* < 0.001; Figure 7D) or unchanged/not detectable within the MC and OL of ALS patients and healthy controls (Figures 7C,D). The protein levels of the proinflammatory chemokine MCP-1 were significantly increased within SC tissue (*p* = 0.011; Figure 7E), but not altered within the MC (*p* = 0.792; Figure 7E), and the OL (*p* = 0.114; Figure 7E) in ALS patients. In addition, MIP-1β (SC: *p* = 0.999) and IL-15 (SC: *p* = 0.184) expression levels were found to be unchanged or undetectable within ALS patients or healthy controls in any of the three regions (Figures 7F,G). Finally, the interferon gamma-induced protein IP10 was significantly reduced within the SC of ALS patients (*p* = 0.011; Figure 7H). No alterations were detectable within the MC (*p* = 0.957) or OL (*p* = 0.229; Figure 7H).

**Figure 8:**
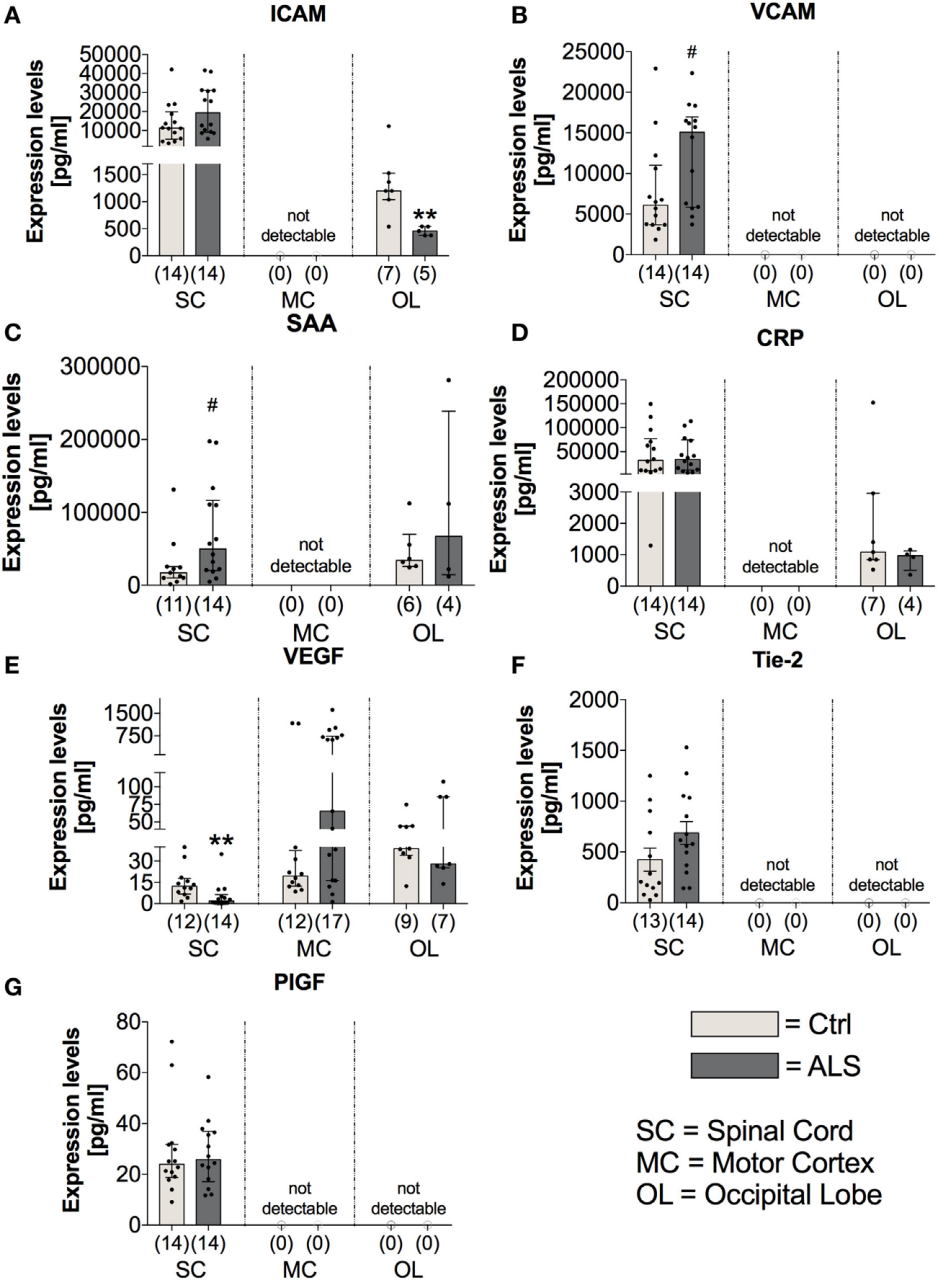
Tissue concentrations of vascular, general inflammation, and angiogenic markers. Amyotrophic lateral sclerosis (ALS) patients exhibited an unchanged expression profile of the vascular and fibrosis-mediating factor ICAM **(A)** within the spinal cord (SC), no detectable amounts in the motor cortex (MC) and a significant reduction in the occipital lobe (OL) **(A)**. For VCAM **(B)**, there was a strong trend toward upregulated amounts in the SC of ALS patients and no detectable levels in the MC and OL **(B)**. The general inflammatory marker SAA **(C)** tended to be upregulated in the SC of ALS patients (SAA, C) or were unchanged or not detectable in the other central nervous system (CNS) regions. The second common marker for inflammation, CRP, was unchanged in all all of the three CNS regions **(D)**. Further, for angiogenic factors including VEGF **(E)**, Tie-2 **(F)**, and PIGF **(G)** only VEGF was significantly reduced in the SC of ALS patients, whereas the other two factors were not altered in the SC and not detectable in any of the two remaining CNS regions. **p < 0.001 vs. controls (Ctrl); # = trend, *p* = 0.057 for VCAM, # = trend, *p* = 0.058 for SAA. Numbers of patients are given in brackets; all parameters were tested for Gaussian distribution using D’Augostino-Pearson omnibus normality test. Two-tailed Mann–Whitney test for ICAM, VCAM, SAA, CRP (OL), VEGF, PIGF, data represent median with interquartile range; two-tailed unpaired Student’s *t*-test for CRP (SC) and Tie-2, data represent mean ± SEM.

Further investigation of the vascular factors ICAM and VCAM revealed unchanged tissue protein levels of ICAM (*p* = 0.178; Figure 8A), whereas a tendency toward an enhanced VCAM protein expression in the SC of ALS patients (*p* = 0.057; Figure 8B). In line, for confirming the hypothesis of an enhanced proinflammatory milieu in ALS patients, analysis of the protein expression levels revealed a trend toward an enhanced amount of SAA in the SC of ALS patients (*p* = 0.058; Figure 8C), but no detectable amounts within the MC, and no changes in the OL (*p* = 0.914). The expression of the general proinflammatory marker CRP was not different in the two groups within the SC (*p* = 0.721) and the OL (*p* = 0.527) and not measurable in the MC (Figure 8D). For the vascular factors VEGF, Tie-2 (Figure 8F) and PIGF (Figure 8G) the only alteration was found for VEGF with significant reduced levels in the SC of ALS patients (*p* = 0.001; Figure 8E).

**Figure 9:**
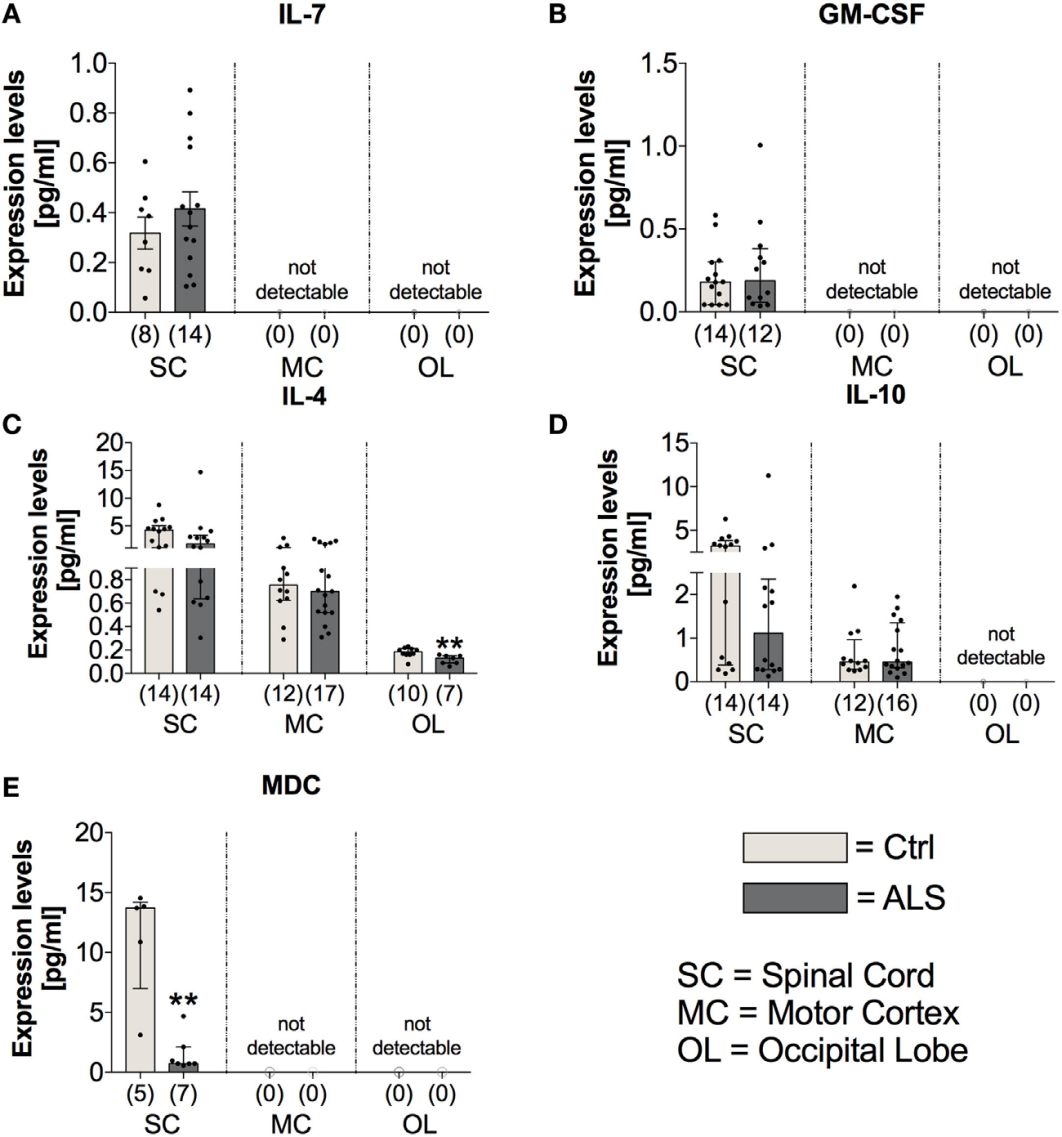
Tissue concentrations of anti-inflammatory cytokines and chemokines. In Amyotrophic lateral sclerosis (ALS) patients tissue, the expression levels of IL-7 **(A)** and GM-CSF **(B)**, both mediating and promoting proliferation of hematopoietic stem cells, were unchanged in the spinal cord (SC) of the two groups and not detectable within the motor cortex (MC) and the occipital lobe (OL). There was a reduced expression of the anti-inflammatory cytokine IL-4 within the OL of ALS patients and the spinal cord (SC) and the MC being unaffected **(C)**. The expression profile of the second anti-inflammatory cytokine IL-10 was unchanged for the two groups within the SC and MC and below detection threshold within the OL **(D)**. The anti-inflammatory chemokine MDC was significantly reduced within the SC of ALS patients compared to healthy controls (Ctrl). There were no detectable amounts in the MC or the OL **(E)**. **p < 0.01 vs. Ctrl. Numbers of patients are given in brackets; All parameters were tested for Gaussian distribution using D’Augostino-Pearson omnibus normality test. Two-tailed Mann–Whitney test for GM-CSF, IL-4, IL-10, and MDC, data represent median with interquartile range; two-tailed unpaired Student’s *t*-test for IL-7, data represent mean ± SEM.

On the other hand, the expression levels of the anti-inflammatory cytokines IL-4 and IL-10 were unchanged within the SC (IL4: *p* = 0.164; IL10: *p* = 0.137) and MC (IL4: *p* = 0.802; IL10: *p* = 0.846) of ALS patients and controls (Figures 9C,D). Within the OL, ALS patients had a significantly reduced amount of IL-4 (*p* = 0.004; Figure 9C), and no expression levels of IL-10 were detectable in any of the two groups (Figure 9D). The anti-inflammatory chemokine MDC was significantly reduced in the SC of ALS patients (*p* = 0.005; Figure 9E).

These results suggest increased abundance of some proinflammatory mediators in ALS SC at end-stage disease.

### ALS Patients Exhibit No Alterations in Factors Mediating the Proliferation/Differentiation of Hematopoietic Stem Cells

In order to investigate possible alterations in the expression of factors mediating the proliferation/differentiation of hematopoietic stem cells and thereby potentially dysregulated compensatory mechanisms for neuronal loss, we analyzed the expression levels of IL-7 and GM-CSF within postmortem tissue samples of the three different CNS areas. Statistical analysis indicated that IL-7 and GM-CSF expression was only detectable in the SC of healthy controls and ALS patients with no differences in their expression profile (IL7: *p* = 0.356; GM-CSF: *p* = 0.781; Figures 9A,B).

### NSC Proliferation Is Shifted towards Quiescence in ALS Patients at Final Disease Stage Paralleled by Reduced Neurogenesis

**Figure 10:**
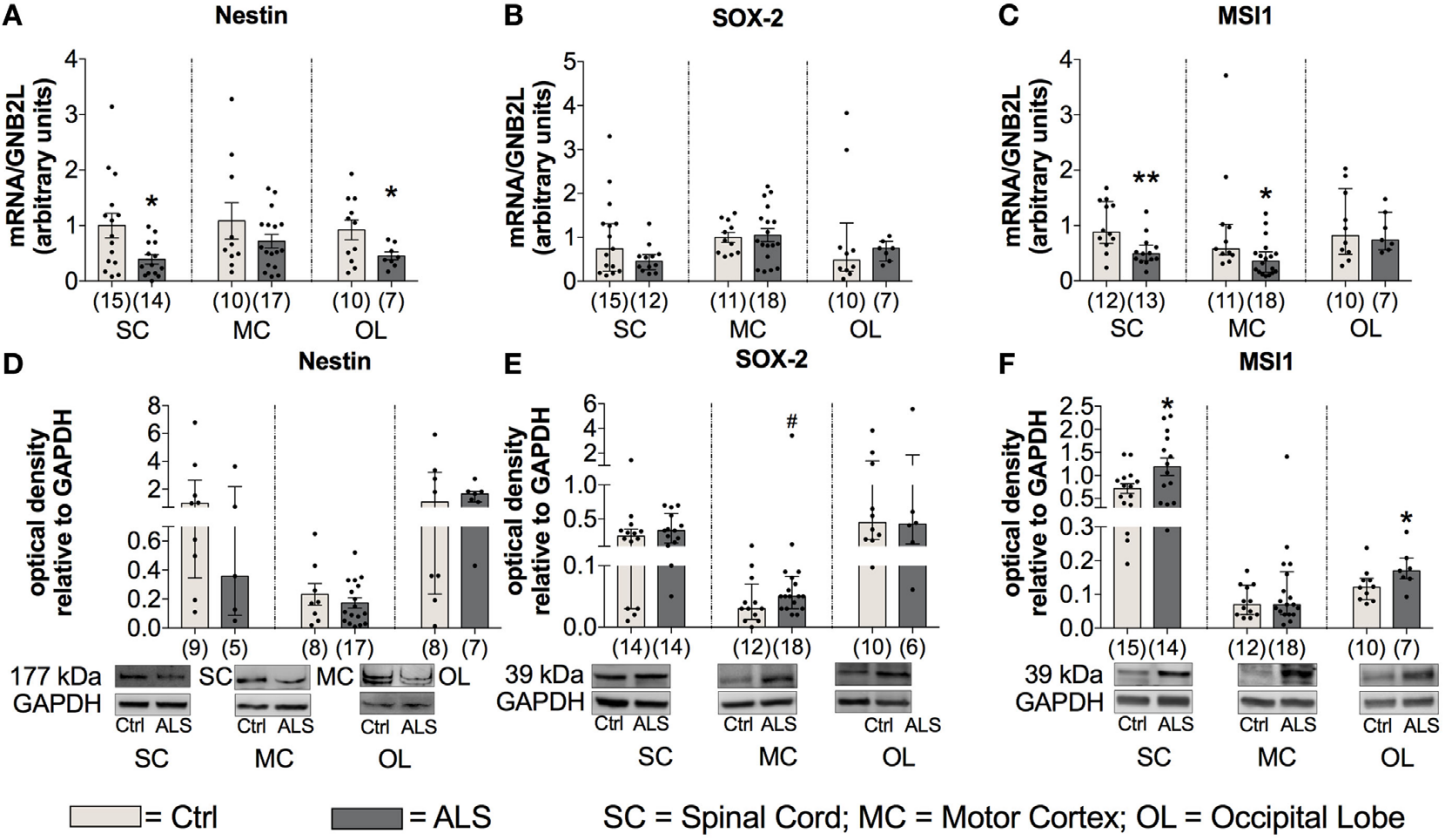
Tissue concentrations of neuronal stem cell markers are reduced in Amyotrophic lateral sclerosis (ALS) patients **(A–F)**. There was a reduced mRNA expression levels of Nestin within the spinal cord (SC) and the occipital lobe (OL) **(A)**, and decreased mRNA amounts of Musashi-1 (MSI1) within the SC and motor cortex (MC) **(C)**. Further, the protein expression of Nestin was unaffected in any of the three central nervous system (CNS) regions **(D)**. ALS patients exhibited a slight trend toward an enhanced SOX-2 protein expression within the motor cortex (MC) **(E)** and significant increased levels of MSI1 in the SC and OL **(F)**. *p < 0.05 vs. controls (Ctrl); **p < 0.01 vs. Ctrl; # = trend, *p* = 0.09. Numbers of patients are given in brackets; all parameters were tested for Gaussian distribution using D’Augostino-Pearson omnibus normality test. Two-tailed Mann–Whitney test for MSI1 mRNA expression, MSI1 protein expression (MC, OL), data represent median with interquartile range. Two-tailed unpaired Student’s *t*-test for Nestin mRNA expression in SC and OL, and for MSI1 protein expression in SC, data represent mean ± SEM. Nestin, SOX-2, and MSI1 protein expression levels were quantified and represented relative to the expression levels of the housekeeping protein GAPDH.

As stated in the introduction, a reduced neurogenesis might promote disease progression by a diminished compensation of neuronal loss. Therefore, to investigate differences in the activity of the adult neuronal niche, postmortem SC, MC, and OL tissue (as an internal control) was analyzed for the expression of the NSC markers Nestin, SOX-2, and Musashi-1. Statistical analysis indicated a reduced Nestin mRNA expression within the SC (*p* = 0.019; Figure 10A) and the OL (*p* = 0.047; Figure 10A) with no changes in the MC (*p* = 0.229; Figure 10A). Sox-2 mRNA expression was unchanged within the SC (*p* = 0.222; Figure 10B), the MC (*p* = 0.809; Figure 10B) or OL (*p* = 0.303; Figure 10B). The mRNA expression levels for the third NSC marker, Musashi-1 (MSI1), were found to be significantly reduced in ALS patients compared to controls within SC (*p* = 0.006; Figure 10C) as well as in MC (*p* = 0.015; Figure 10C), but unchanged for OL (*p* = 0.983; Figure 10C).

To get more information about the activity of NSCs, the protein expression was also assessed for the three different markers within the three CNS areas. Statistical analysis indicated unchanged Nestin protein levels in ALS patients compared to healthy controls in any of the three different CNS regions (SC: *p* = 0.298; MC: *p* = 0.424; OL: *p* = 0.694; Figure 10D). Further, the analysis of Sox-2 protein expression revealed a trend toward an enhanced amount in the MC of ALS patients compared to healthy controls (*p* = 0.090; Figure 10E). Finally, an enhanced protein level of MSI1 in SC (*p* = 0.034; Figure 10F) was detected, no differences were found for MC (*p* = 0.668; Figure 10F), and a significantly enhanced level within OL (*p* = 0.043; Figure 10F).

Taken together, these results suggest a reduced/inhibited activity of the adult neurogenic niche in postmortem tissue of ALS patients compared to controls.

**Figure 11:**
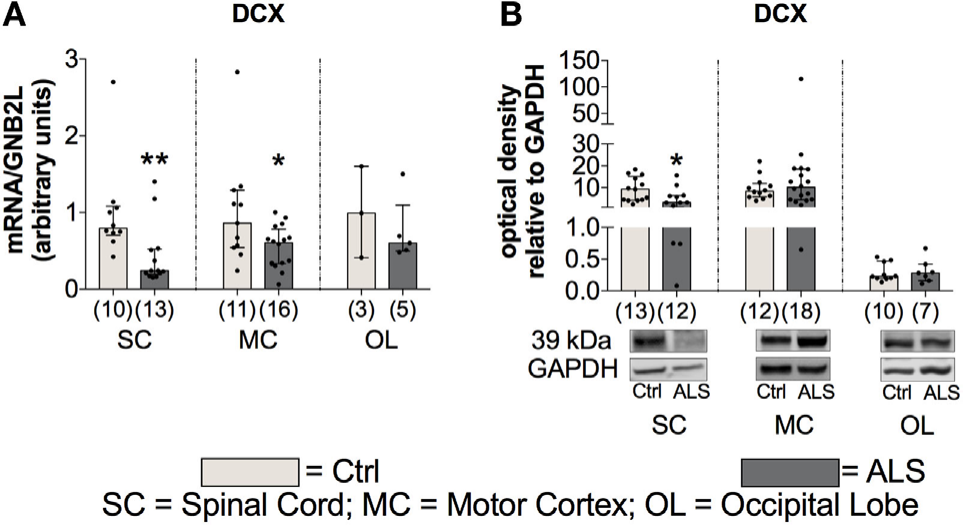
Tissue concentration of neurogenesis marker is reduced in Amyotrophic lateral sclerosis (ALS) patients within the spinal cord (SC) and motor cortex (MC) indicated by reduced DCX mRNA and protein expression in the SC and a decreased DCX mRNA expression within the MC **(A, B)**. Expression levels within the occipital lobe (OL) were unchanged on mRNA and protein level. *p < 0.05 vs. controls (Ctrl); **p < 0.01 vs. Ctrl. Numbers of patients are given in brackets; All parameters were tested for Gaussian distribution using D’Augostino-Pearson omnibus normality test. Two-tailed Mann–Whitney test, data represent median with interquartile range. DCX protein expression levels were quantified and represented relative to the expression levels of the housekeeping protein GAPDH.

To further investigate whether this reduced activity of the adult neurogenic niche results in a downregulated neurogenesis, we determined the mRNA as well the protein expression levels of the well described neurogenesis marker doublecortin (DCX). Here, statistical analysis revealed a reduced DCX mRNA expression within the SC (*p* = 0.004; Figure 11A), and the MC (*p* = 0.047; Figure 11A), but not within the OL (*p* = 0.786; Figure 11A). In addition, DCX protein expression was also significantly reduced within the SC of ALS patients compared to controls (*p* = 0.035; Figure 11B), but unchanged within the MC (*p* = 0.851; Figure 11B) and the OL (*p* = 0.813; Figure 11B).

These results suggest that in addition to a possible reduced/inhibited activity of the adult neurogenic niche, ALS patients might have also a reduced neurogenesis compared to controls.

### Fibrotic Activity Is Enhanced at Final Disease Stage of ALS Patients

**Figure 12:**
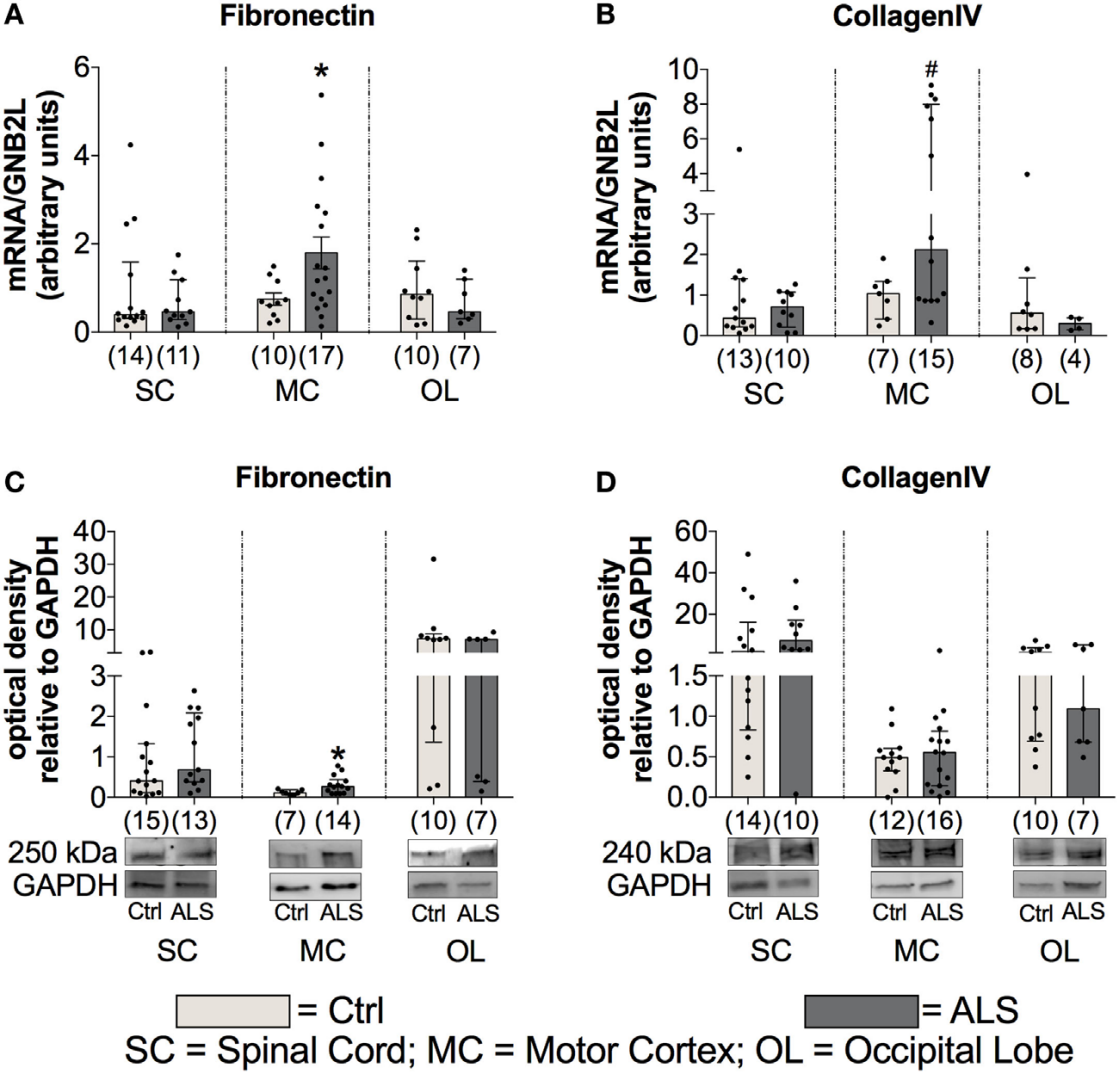
Tissue concentrations of fibrotic markers. Postmortem tissue of Amyotrophic lateral sclerosis (ALS) patients had an increased Fibronectin mRNA **(A)** and protein **(C)** expression levels in the motor cortex (MC). Further, there was a strong trend toward an enhanced CollagenIV expression in the MC of ALS patients **(D)**. CollagenIV mRNA levels were unchanged in all three CNS levels **(B)**. The amounts of both fibrotic markers were unchanged in spinal cord (SC) and the occipital lobe (OL) of ALS patients and healthy controls (Ctrl). *p < 0.05 vs. Ctrl; # = trend, *p* = 0.06 vs. Ctrl. Numbers of patients are given in brackets; all parameters were tested for Gaussian distribution using D’Augostino-Pearson omnibus normality test. Two-tailed Mann–Whitney test for Fibronectin mRNA (SC, OL), protein expression (SC, MC, OL), collagen IV mRNA (SC, MC, OL), and collagen IV protein expression (SC, MC, OL), data represent median with interquartile range. Two-tailed unpaired Student’s *t*-test for fibronectin mRNA expression (MC), data represent mean ± SEM. Fibronectin and collagen IV protein expression levels were quantified and represented relative to the expression levels of the housekeeping protein GAPDH.

Finally, to investigate fibrotic processes within postmortem SC, MC, and OL tissue, potentially promoting ALS disease progression, we analyzed the expression levels of two major components of ECM, fibronectin and collagen IV. Statistical analysis indicated no alterations in fibronectin mRNA expression levels within the SC (*p* = 0.990; Figure 12A) and the OL (*p* = 0.601; Figure 12A), but enhanced within the MC (*p* = 0.041; Figure 12A) of ALS patients compared to healthy controls. Similarly, fibronectin protein expression was unchanged within the SC (*p* = 0.580; Figure 12C), and the OL (*p* = 0.403; Figure 12C), but significantly increased within the MC (*p* = 0.039; Figure 12C) of ALS patients. Increased collagen IV mRNA expression in MC tissue was borderline significant (*p* = 0.058; Figure 12B), and unaltered in the SC (*p* > 0.999; Figure 12B), and the OL (*p* = 0.265; Figure 12B). Collagen IV protein expression remained unchanged in the SC (*p* = 0.312; Figure 12D), MC (*p* = 0.793; Figure 12A), and OL (*p* = 0.962; Figure 12D) of ALS patients compared to healthy controls.

These results give evidence for enhanced fibrotic processes in ALS patients compared to healthy controls, which might further facilitate disease progression.

## Discussion

**Figure 13:**
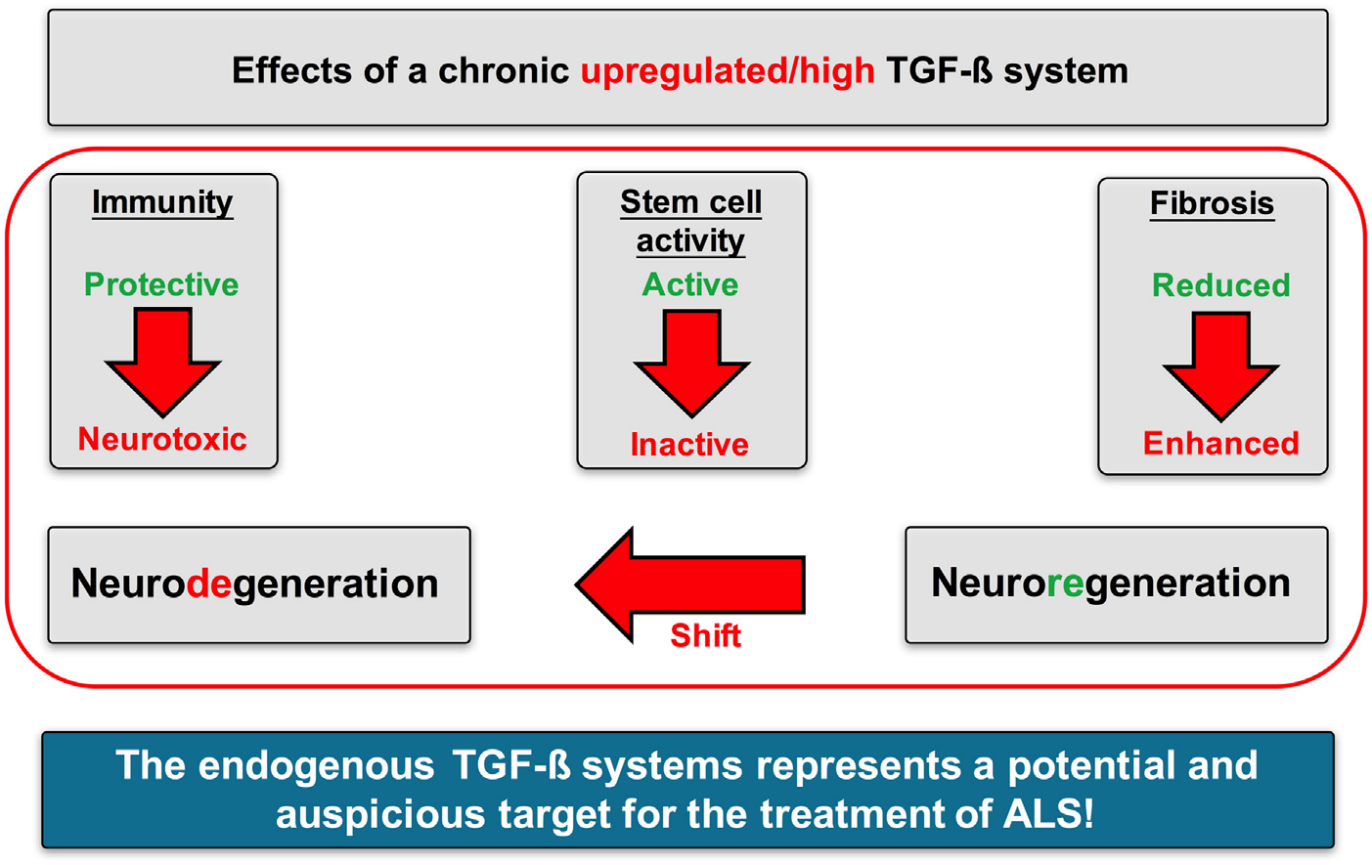
Proposed mechanisms of action of TGF-β signaling in Amyotrophic lateral sclerosis (ALS). A chronically upregulated TGF-β system activity promoted the imbalance of neurodegenerative and neuroregenerative processes favoring neuronal loss by (i) shifting the immune response toward a proinflammatory neurotoxic and neurodestructive milieu, (ii) shifting the activation state of neuronal stem cells toward an arrested/inhibited phenotype, and (iii) promoting fibrotic activity at the side of neuroinflammation and neuronal loss.

In the present study, we could create some evidence from human data that an enhanced TGF-β system activity may critically mediate the imbalance of neuroregenerative and neurodegenerative processes in ALS (Figure 13).

The analysis of patient sera and postmortem SC tissue samples suggests an enhanced “neurodestructive” immune profile in ALS patients compared to healthy controls, reflected by increased expression of proinflammatory versus unchanged or reduced levels of anti-inflammatory cytokines, which is in line with a previous study (49). Further, sera of ALS patients displayed enhanced levels of endothelial and vascular factors, indicating an enhanced proinflammatory, proangiogenic, and profibrotic activity.

At first glance, these results seem to contrast with concomitant enhanced TGF-β serum and postmortem tissue levels in ALS patients (Figures 1 and 5). The most prominent effect of TGF-β within the immune system, is its ability to stimulate maturation of CD4^+^CD25^+^FoxP3^+^ T cells (T_Regs_) (50). As a well-established factor in ALS progression, T lymphocytes infiltrate the CNS at the level of the SC and along the vessels within the precentral gyrus extending into the areas of neuronal injury (51–54). Animal models have demonstrated that CD4^+^ T lymphocytes shift the profile of macrophages and activated microglia cells toward an anti-inflammatory neuroprotective M2-like phenotype that further induces T_Regs_. Enhanced T_Reg_ levels in the initial and relatively stable phase of ALS progression diminish the activity of CD4^+^CD25^−^ effector T cells (55–57). However, a recent study has demonstrated a continuous decline of T_Regs_ throughout the course of disease leading to a negative correlation with ALS progression rate (58). T_Regs_ isolated from ALS patients, phenotypically identical to those from healthy individuals, are functionally impaired in their ability to suppress effector T cell functions. Importantly, the *in vitro* expansion of these autologous cells restored their immune regulatory activity, indicating a complete exhaustion due to a persistent and continuous stimulation (59). Therefore, enhanced TGF-β serum levels (Figure 1) might reflect an autocrine and compensatory mechanism to fight the neurotoxic Th1-mediated immune response. In areas already affected by inflammation and in the presence of proinflammatory factors, TGF-β exerts strong opposite effects. In combination with high levels of IL-6, TGF-β induces T cells to differentiate into non-regulatory phenotypes such as proinflammatory Th17 effectors (60).

*In vitro* experiments have demonstrated that TGF-β promotes MCP-1 release *per se* and massively amplifies MCP-1 release upon costimulation with IL-1β [(61), Figures 7D,E]. Both, MCP-1 and IL-1β, represent prominent proinflammatory chemoattractants for astrocytes, another important source of enhanced TGF-β levels in ALS patients and transgenic mice. Excessive astrocytic TGF-β levels were shown to suppress the neuroprotective anti-inflammatory responses by microglia and T cells, and thereby accelerate disease progression in ALS mice (62).

In addition to neuronal loss, reduced neurogenesis and suppressed NSC activity represent a second hallmark of neurodegeneration. In the adult neurogenic niche, TGF-β acts as a double-edged sword, by favoring neuronal differentiation and survival in normal brain, and preferring cell cycle arrest and stem cell quiescence in the damaged brain (39, 42, 63–66). These different effects relate to dose-dependent effects of TGF-β (Figures 1 and 5), that are well-established in hematological paradigms: at low doses, TGF-β stimulates myeloid, but inhibits lymphoid hematopoietic stem cell proliferation. In contrast, at high doses, TGF-β inhibits proliferation, irrespective of hematopoietic stem cell differentiation subtype, and induces quiescence (38). Although we found enhanced serum levels of IL-7 in ALS patients and IL-7 stimulates proliferation and differentiation of hematopoietic stem cells in healthy individuals (67, 68), the amount of CD34^+^ hematopoietic stem cells was unchanged (Figure 4). Taken together these findings support the hypothesis of stem cell quiescence in the ALS bone marrow. In line with these findings, *in vivo* studies in transgenic Huntington’s disease rats have demonstrated elevated TGF-β signaling within the SGZ, reducing proliferation of NSCs and thereby decreasing DCX-positive cells and neurogenesis (69). Reduced mRNA levels of the NSC markers MSI-1 (SC, MC) and Nestin (SC, OL), enhanced MSI-1 protein levels within the SC and a trend toward increased Sox-2 within the MC of ALS patients indicate protein accumulation, and support the theory of a shift toward an inhibited/arrested activity of NSCs (Figure 10). The reduced DCX mRNA (SC, MC) and protein (SC) expression patterns in the present analysis (Figure 11) indicate a severely diminished neurogenic niche activity.

In addition, TGF-β could affect hippocampal neurogenesis indirectly *via* immune interactions (70, 71). Thereby, TNFα exerts inhibiting (72, 73) or promoting properties, depending on receptor subtype binding, with TNFR1 negatively (74–76), and TNFR2 positively influencing hippocampal neurogenesis (77, 78). The effects of IL-1β (massively in ALS-SC, Figure 7D) on adult neurogenesis are detrimental (37, 79–82), but reversible (83, 84). The authors are aware that adult human neurogenesis was exclusively shown within the SGZ and the SVZ so far. However, adult NSCs can be isolated also from human white matter tissue (31). Our data show that NSCs are also detectable in human SC. This fact indicates that neuronal repair may not only occur in SGZ or SVZ, but also in human SC, confirming *in vivo* studies in mice, rats, and primates (32–34).

Finally, fibrotic activities represent a third hallmark of neurodegeneration. Enhanced fibrosis within the CNS can be a consequence of infections, parasite infestations, and neuronal injury (41). In most organs, protracted inflammation due to ongoing infectious or toxic tissue destruction increases the amount of ECM. The strong trend toward enhanced CollagenIV mRNA and significantly increased Fibronectin mRNA, and protein levels within postmortem MC tissue of ALS patients (Figure 12), indicate an increased profibrotic activity. Again, IL-6, IL-1β, and TNFα (Figure 7), all secreted by microglia or infiltrating immune cells, represent the most prominent cytokines to aggravate fibrosis (85). Among those cytokines, IL-1β and specifically TGF-β act directly upon fibroblasts or their respective precursor cells, inducing a profibrotic phenotype (43). The chemokine TARC, mostly produced by Th2 cells and enhanced in sera of our ALS patients (Figure 2), was shown to be predominantly expressed in epithelial cells, following experimental-induced pulmonary fibrosis in mice, but also in human idiopathic pulmonary fibrosis lung tissue. Neutralization of this chemokine results in an attenuation of bleomycin-induced pulmonary fibrosis in mice (86). Therefore, TGF-β might promote fibrotic effects by its initial activation of Th2/M2/T_Reg_ immune effects at an earlier stage of ALS progression. However, TGF-β drives the fibrotic scar formation by inhibiting deposition of fibrotic scar tissue (87), affecting pericytes, myofibroblasts and fibroblasts (41), and promoting expression of profibrotic growth factors including CTGF, FGF-2, and PDGF (88–91) in later disease.

The authors are aware of the seemingly marginal differences of TGF-β ligand levels within sera and CNS tissue lysates between ALS patients and healthy controls. Therefore, animal models might be employed to prove the hypothesis that slight alterations of TGF-β system activity suffice to initiate and promote disease progression in ALS. However, studies using antagonists or knock out animals simulate extreme situations with either highly over- or downregulated systems compared to physiological situations. We therefore consider our human data to be more reflective to TGF-β system alterations. A marginally but constantly enhanced TGF-β signaling was sufficient to induce strong physiological alterations (92): responses to different TGF-β doses were extremely time dependent with a graded short-term signaling, and a switch-like long-term response. Therefore, small alterations in TGF-β doses within a certain range lead to biologically relevant changes in the signal and the adjacent intracellular response.

Interestingly, it is important to point out that we were able to observe specific changes in different CNS tissues including the MC (surrounding milieu of upper motor neurons), the SC (surrounding milieu of motor neurons), and the OL (relatively unaffected in ALS). The specific immunological, neurogenic, and fibrotic alterations described and discussed in the previous paragraphs might indicate potential “hot spots” of ALS disease burden. This may reflect the hypothesis for initial local foci that continuously expand to adjacent areas. This idea is in line with the model of Braak, describing a consistent spread from affected areas during different stages of disease burden (93). Our observed serum sample (peripheral, in life changes) alterations of ALS patients, which are comparable to those described for postmortem CNS tissues, further support the idea of specific physiological/immunological changes, associated with disease spreading during different disease stages and therefore potentially mediating and driving ALS progression.

Taken together, our data support the hypothesis that the TGF-β system may represent a critical factor for disease modulation in ALS. In context with specific cytokines, a persistently enhanced TGF-β signaling seems to result in an imbalance of neuroregeneration and neurodegeneration. Neurodegenerative processes occur more often due to a profound proinflammatory immune response, an inhibited/arrested adult neurogenic niche activity, and an increased profibrotic status. Therefore, the TGF-β system may represent a very promising target for ALS.

## Ethics Statement

The study for the analysis of human serum samples was carried out with the recommendations of the ethics committee at the University of Regensburg with written informed consent from all subjects. All subjects gave written informed consent in accordance with the Declaration of Helsinki. The study for the analysis of human postmortem tissue samples was carried out with the recommendations of different ethical votes from participating universities including the MND-network votes with written informed consent from all subjects. All subjects gave written informed consent in accordance with the Declaration of Helsinki.

## Author Contributions

SP: study design, protein expression *via* immunefluorescence staining and data analysis, writing, and drafting the manuscript. EZ: protein expression analysis *via* Western blot and MSD assay, mRNA expression analysis *via* qRT-PCR. SK: protein expression analysis *via* Western blot, revision of manuscript. SI: editing of the human blood/serum samples, determination of CD34^+^ hematopoietic stem cells, and revision of manuscript. RH: protein expression analysis *via* Western Blot, mRNA expression analysis *via* qRT-PCR. SJ: protein expression analysis *via* Western Blot, revision of manuscript. SP: providing human postmortem CNS tissue, revision of manuscript. LA: study design, revision of manuscript. DT: providing human postmortem CNS tissue, revision of manuscript. AH: providing human postmortem CNS tissue, revision of manuscript. JW: providing human postmortem CNS tissue, revision of manuscript. T-HB: study design, revision of manuscript. UB: study design, revision of manuscript, and holder of BMBF GO-Bio Grant.

## Conflict of Interest Statement

UB and LA are partners at NeuroVision Pharma GmbH, which holds intellectual property on TGF-beta receptor modulators. All other authors declare that they do not have any conflicts of interest.

## Supplementary Material

Figure S1 Whole Blots for TGF-β1 **(A)**, TGF-β2 **(B)**, TGF-βRI **(C)**, and TGF-βRII **(D)** within human postmortem spinal cord tissue. Figures show protein standard ladder, whole blot for the respective analytes and the whole blot for the house-keeper GAPDH. Red squares indicate the representative blots shown within the main manuscript.

Figure S2 Whole Blots for Nestin **(A)**, Sox-2 **(B)**, Msi-1 **(C)**, and DCX **(D)** within human postmortem spinal cord tissue. Figures show protein standard ladder, whole blot for the respective analytes and the whole blot for the house-keeper GAPDH. Red squares indicate the representative blots shown within the main manuscript.

Figure S3 Whole Blots for fibronectin **(A)** and CollagenIV **(B)** within human postmortem spinal cord tissue. Figures show protein standard ladder, whole blot for the respective analytes and the whole blot for the house-keeper GAPDH. Red squares indicate the representative blots shown within the main manuscript.

Figure S4 Whole Blots for TGF-β1 **(A)**, TGF-β2 **(B)**, TGF-βRI **(C)**, and TGF-βRII **(D)** within human postmortem motor cortex tissue. Figures show protein standard ladder, whole blot for the respective analytes and the whole blot for the house-keeper GAPDH. Red squares indicate the representative blots shown within the main manuscript.

Figure S5 Whole Blots for Nestin **(A)**, Sox-2 **(B)**, Msi-1 **(C)**, and DCX **(D)** within human postmortem motor cortex tissue. Figures show protein standard ladder, whole blot for the respective analytes and the whole blot for the house-keeper GAPDH. Red squares indicate the representative blots shown within the main manuscript.

Figure S6 Whole Blots for fibronectin **(A)** and CollagenIV **(B)** within human postmortem motor cortex tissue. Figures show protein standard ladder, whole blot for the respective analytes and the whole blot for the house-keeper GAPDH. Red squares indicate the representative blots shown within the main manuscript.

Figure S7 Whole Blots for TGF-β1 **(A)**, TGF-β2 **(B)**, TGF-βRI **(C)**, and TGF-βRII **(D)** within human postmortem occipital lobe tissue. Figures show protein standard ladder, whole blot for the respective analytes and the whole blot for the house-keeper GAPDH. Red squares indicate the representative blots shown within the main manuscript.

Figure S8 Whole Blots for Nestin **(A)**, Sox-2 **(B)**, Msi-1 **(C)**, and DCX **(D)** within human postmortem occipital lobe tissue. Figures show protein standard ladder, whole blot for the respective analytes and the whole blot for the house-keeper GAPDH. Red squares indicate the representative blots shown within the main manuscript.

Figure S9 Whole Blots for fibronectin **(A)** and CollagenIV **(B)** within human postmortem occipital lobe tissue. Figures show protein standard ladder, whole blot for the respective analytes and the whole blot for the house-keeper GAPDH. Red squares indicate the representative blots shown within the main manuscript.

Table S1 Cytokines/chemokines analyzed in human serum samples of healthy controls and ALS patients but not shown as graphs within the manuscript.

Table S2 Cytokines/chemokines analyzed in human postmortem samples of healthy controls and ALS patients but not shown as graphs within the manuscript.
