# Supplementary figures and images for: The TGF-β System As a Potential Pathogenic Player in Disease Modulation of Amyotrophic Lateral Sclerosis
Source: Front Neurol. 2017 Dec 15;8:669. doi: 10.3389/fneur.2017.00669 (PMC5736544; doi:10.3389/fneur.2017.00669)

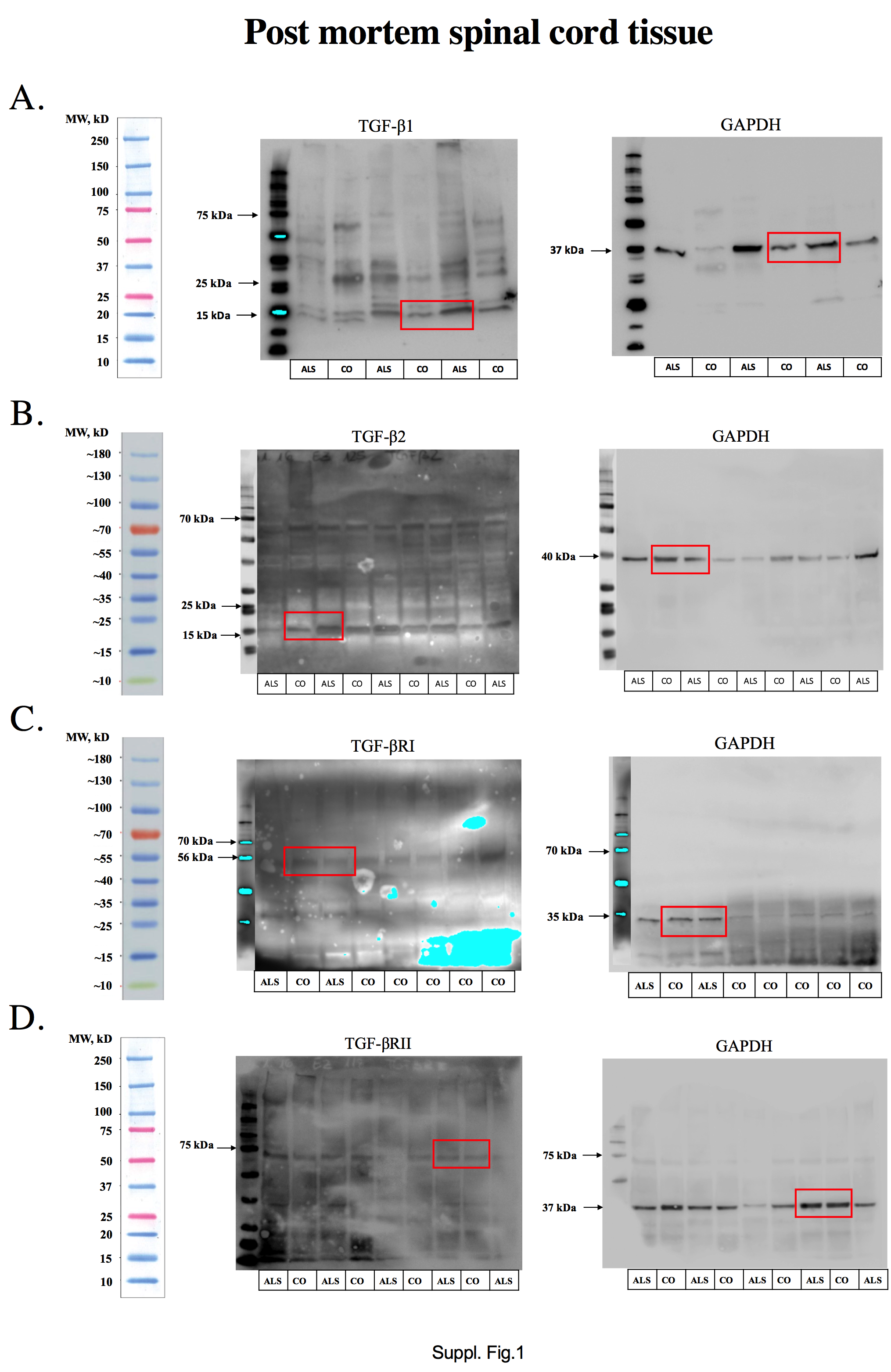

Supplement: Figure S1 — Whole Blots for TGF-β1 (A), TGF-β2 (B), TGF-βRI (C), and TGF-βRII (D) within human postmortem spinal cord tissue. Figures show protein standard ladder, whole blot for the respective analytes and the whole blot for the house-keeper GAPDH. Red squares indicate the representative blots shown within the main manuscript. [file Image_1.tiff]

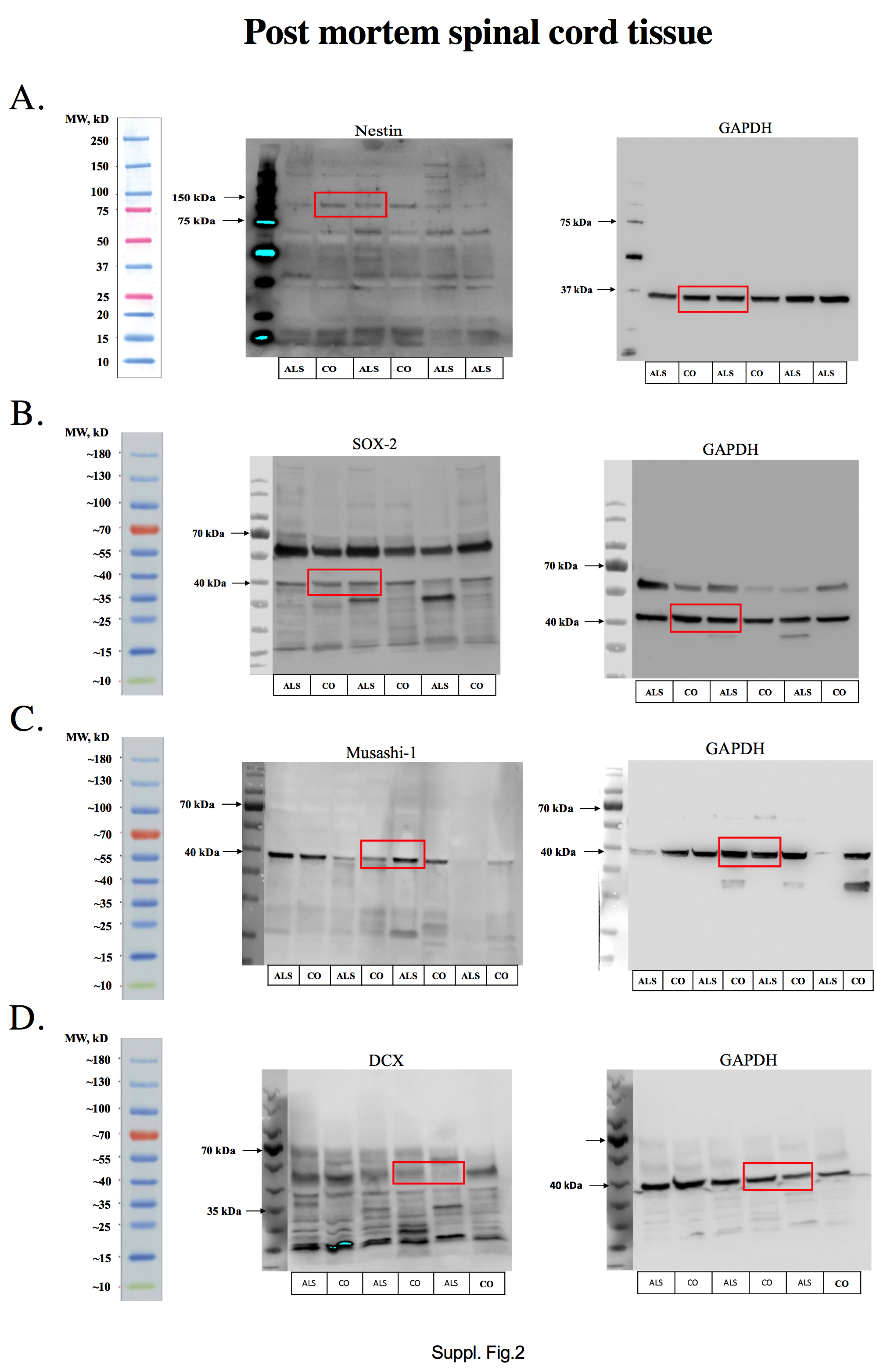

Supplement: Figure S2 — Whole Blots for Nestin (A), Sox-2 (B), Msi-1 (C), and DCX (D) within human postmortem spinal cord tissue. Figures show protein standard ladder, whole blot for the respective analytes and the whole blot for the house-keeper GAPDH. Red squares indicate the representative blots shown within the main manuscript. [file Image_2.tiff]

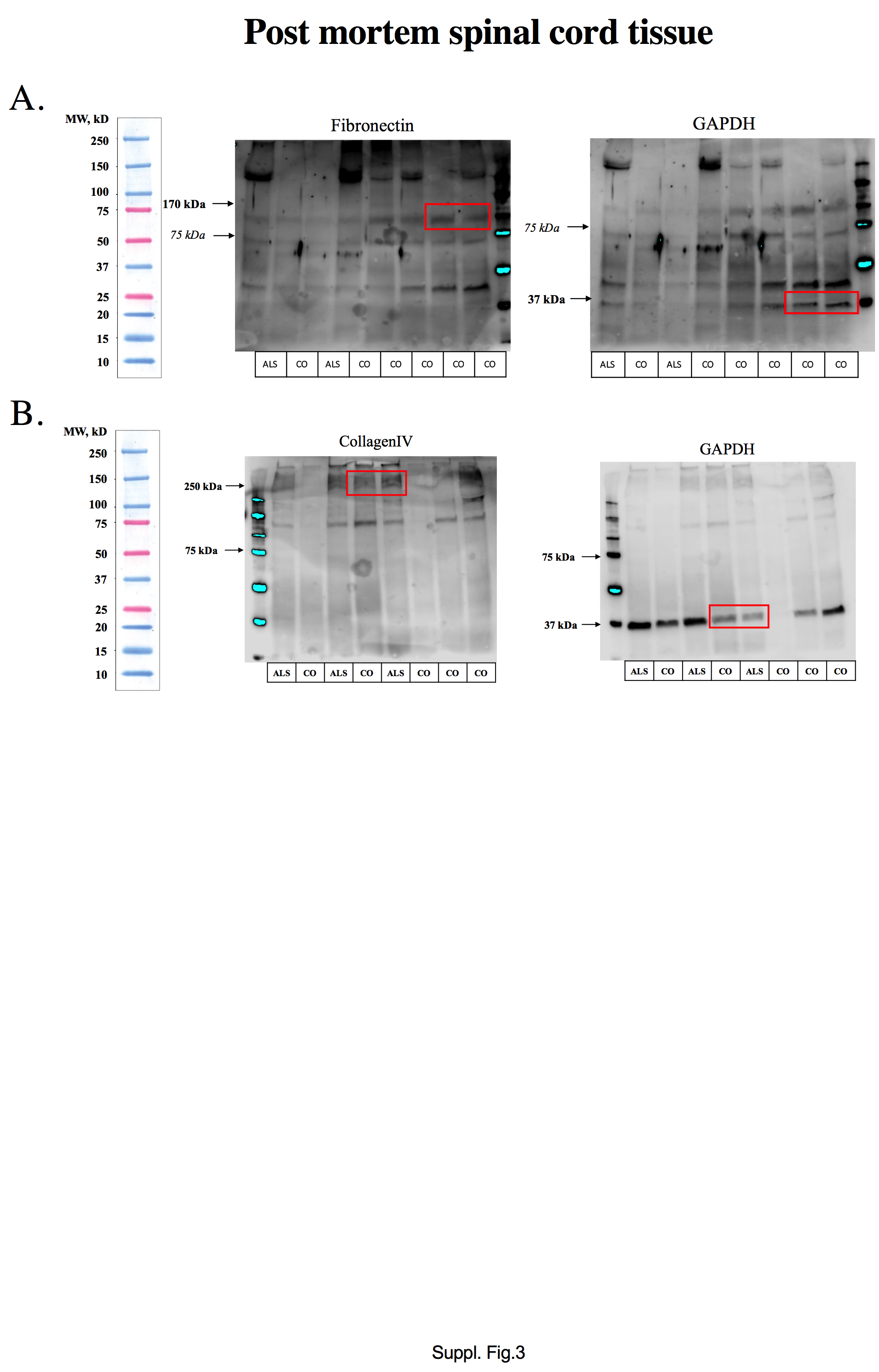

Supplement: Figure S3 — Whole Blots for fibronectin (A) and CollagenIV (B) within human postmortem spinal cord tissue. Figures show protein standard ladder, whole blot for the respective analytes and the whole blot for the house-keeper GAPDH. Red squares indicate the representative blots shown within the main manuscript. [file Image_3.tiff]

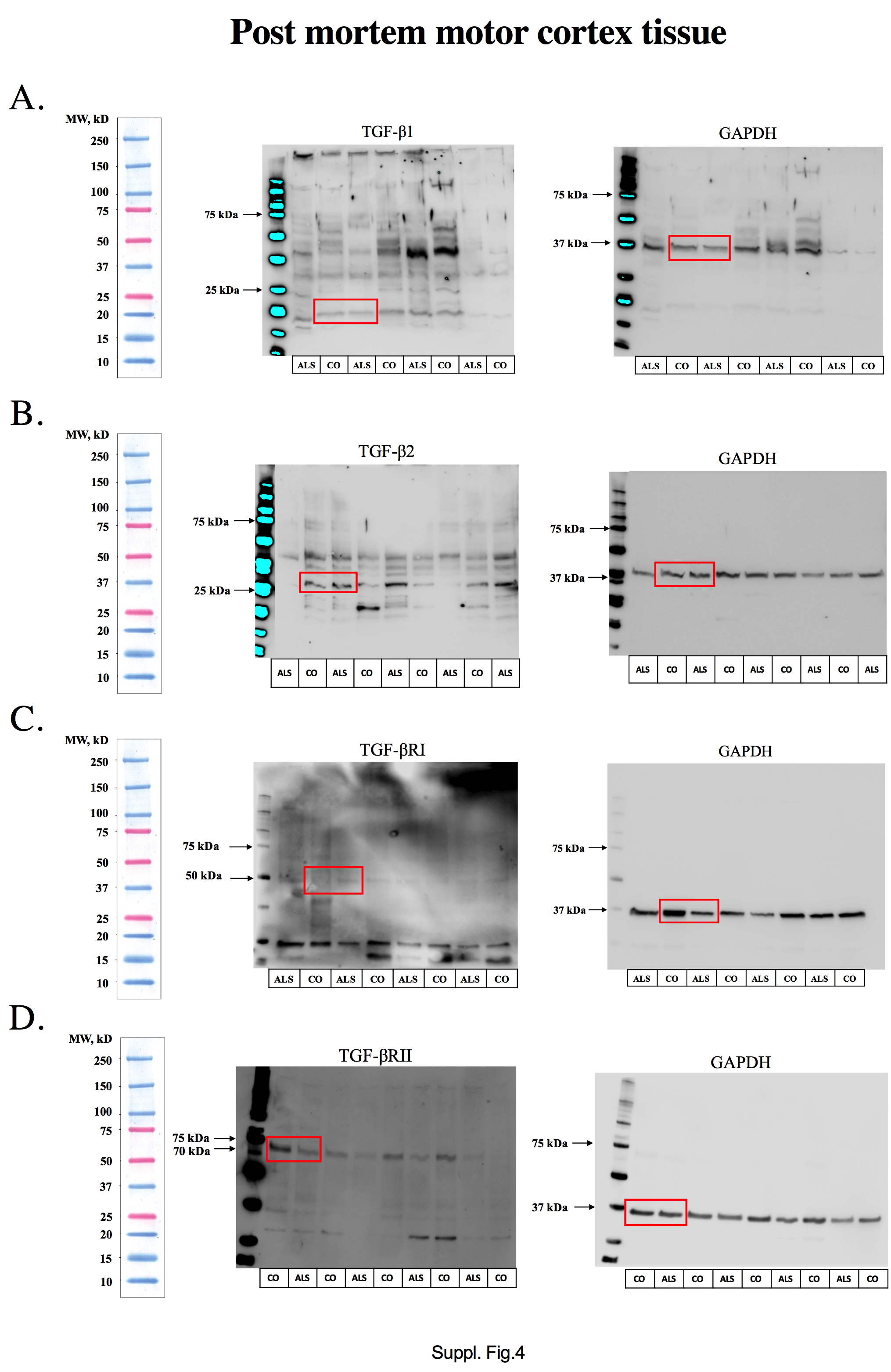

Supplement: Figure S4 — Whole Blots for TGF-β1 (A), TGF-β2 (B), TGF-βRI (C), and TGF-βRII (D) within human postmortem motor cortex tissue. Figures show protein standard ladder, whole blot for the respective analytes and the whole blot for the house-keeper GAPDH. Red squares indicate the representative blots shown within the main manuscript. [file Image_4.tiff]

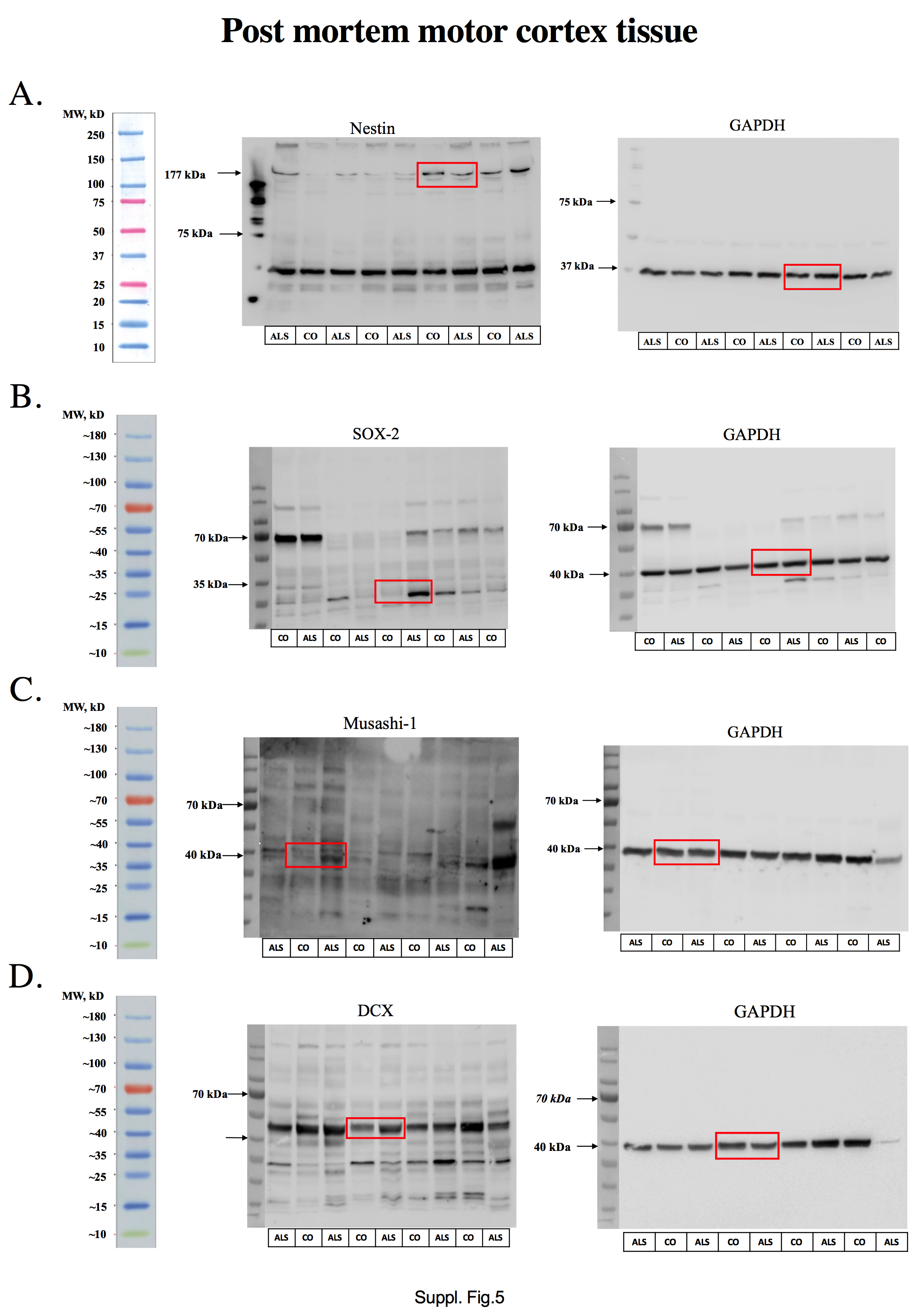

Supplement: Figure S5 — Whole Blots for Nestin (A), Sox-2 (B), Msi-1 (C), and DCX (D) within human postmortem motor cortex tissue. Figures show protein standard ladder, whole blot for the respective analytes and the whole blot for the house-keeper GAPDH. Red squares indicate the representative blots shown within the main manuscript. [file Image_5.tiff]

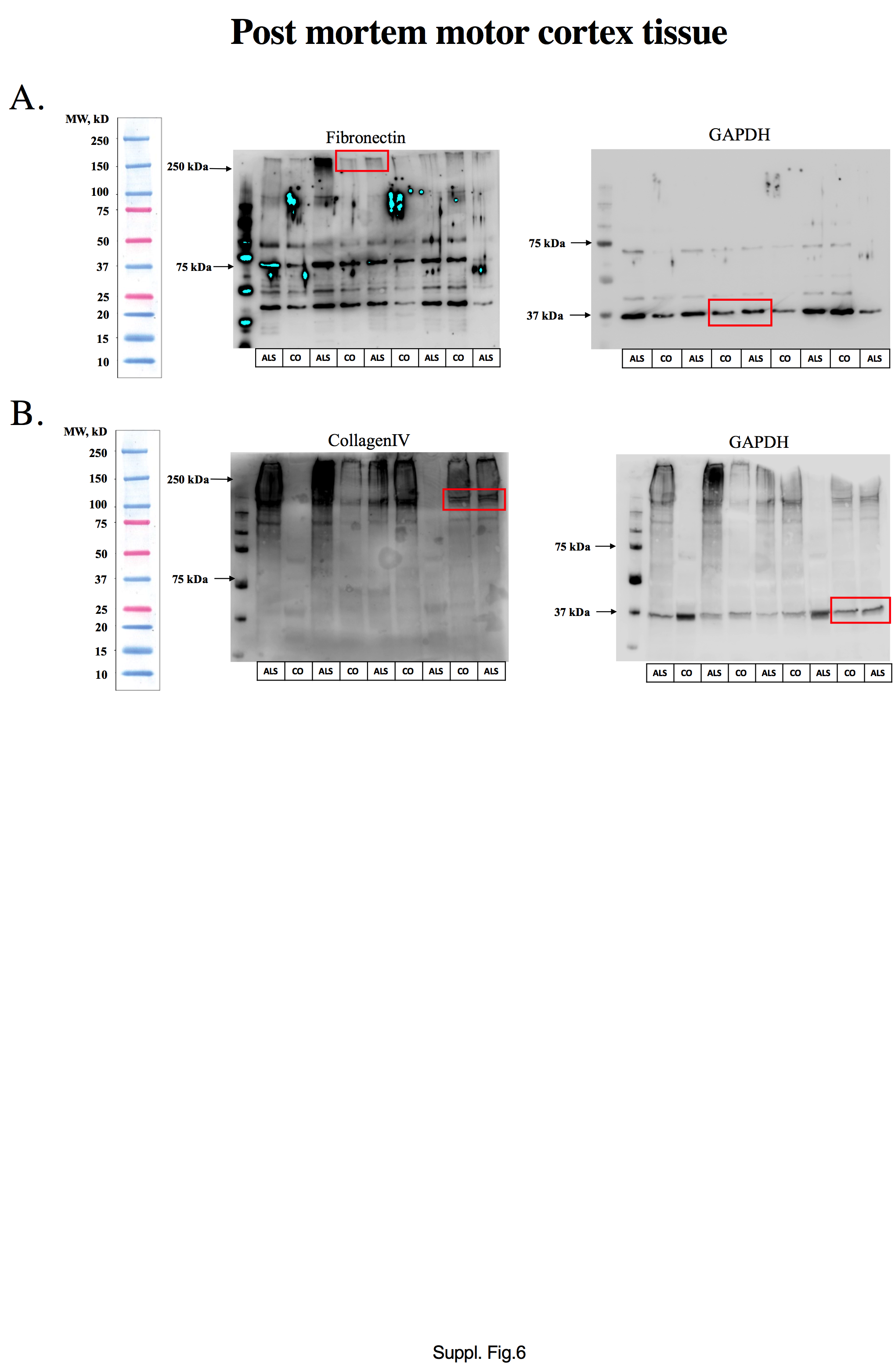

Supplement: Figure S6 — Whole Blots for fibronectin (A) and CollagenIV (B) within human postmortem motor cortex tissue. Figures show protein standard ladder, whole blot for the respective analytes and the whole blot for the house-keeper GAPDH. Red squares indicate the representative blots shown within the main manuscript. [file Image_6.tiff]

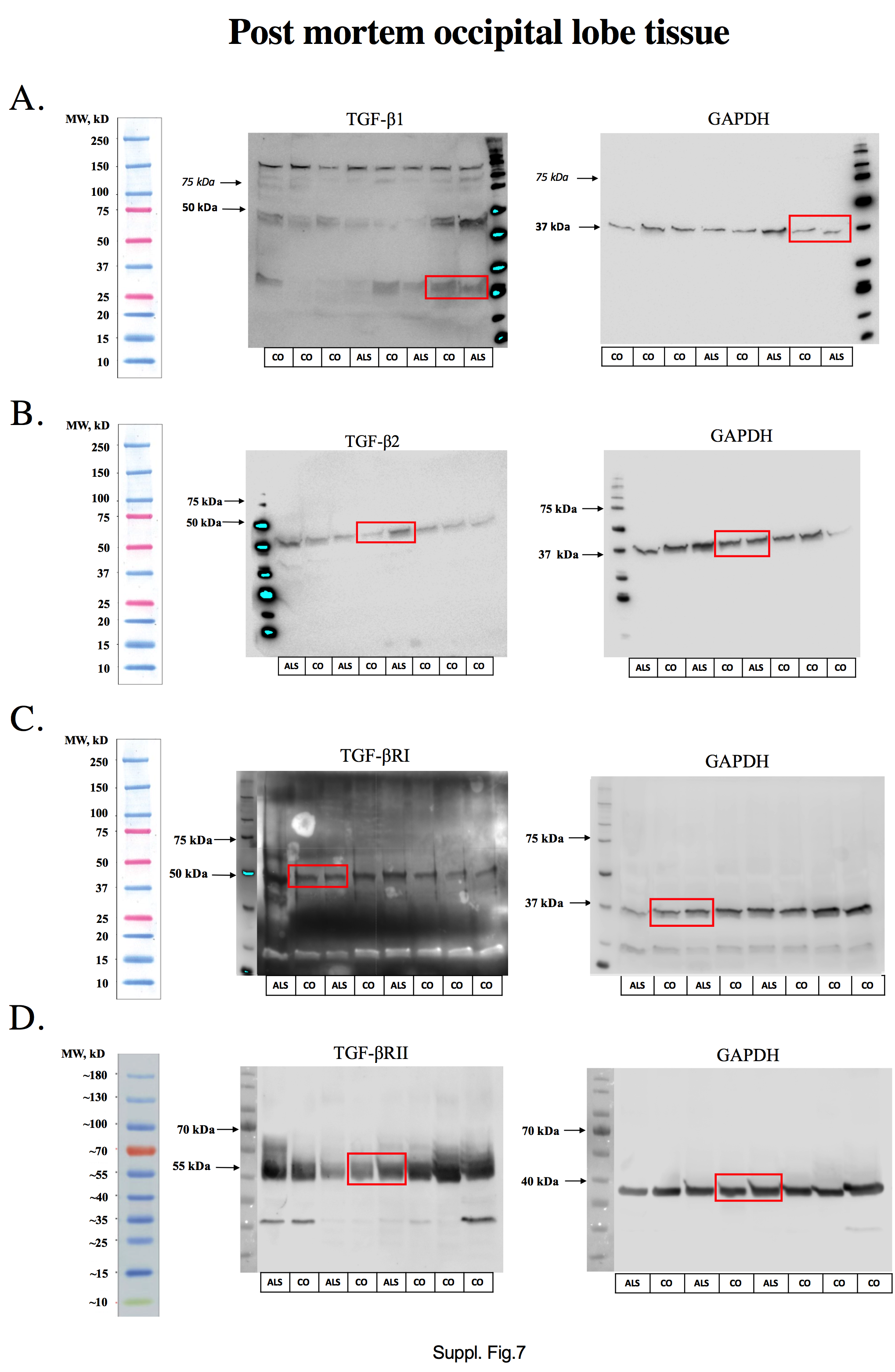

Supplement: Figure S7 — Whole Blots for TGF-β1 (A), TGF-β2 (B), TGF-βRI (C), and TGF-βRII (D) within human postmortem occipital lobe tissue. Figures show protein standard ladder, whole blot for the respective analytes and the whole blot for the house-keeper GAPDH. Red squares indicate the representative blots shown within the main manuscript. [file Image_7.tiff]

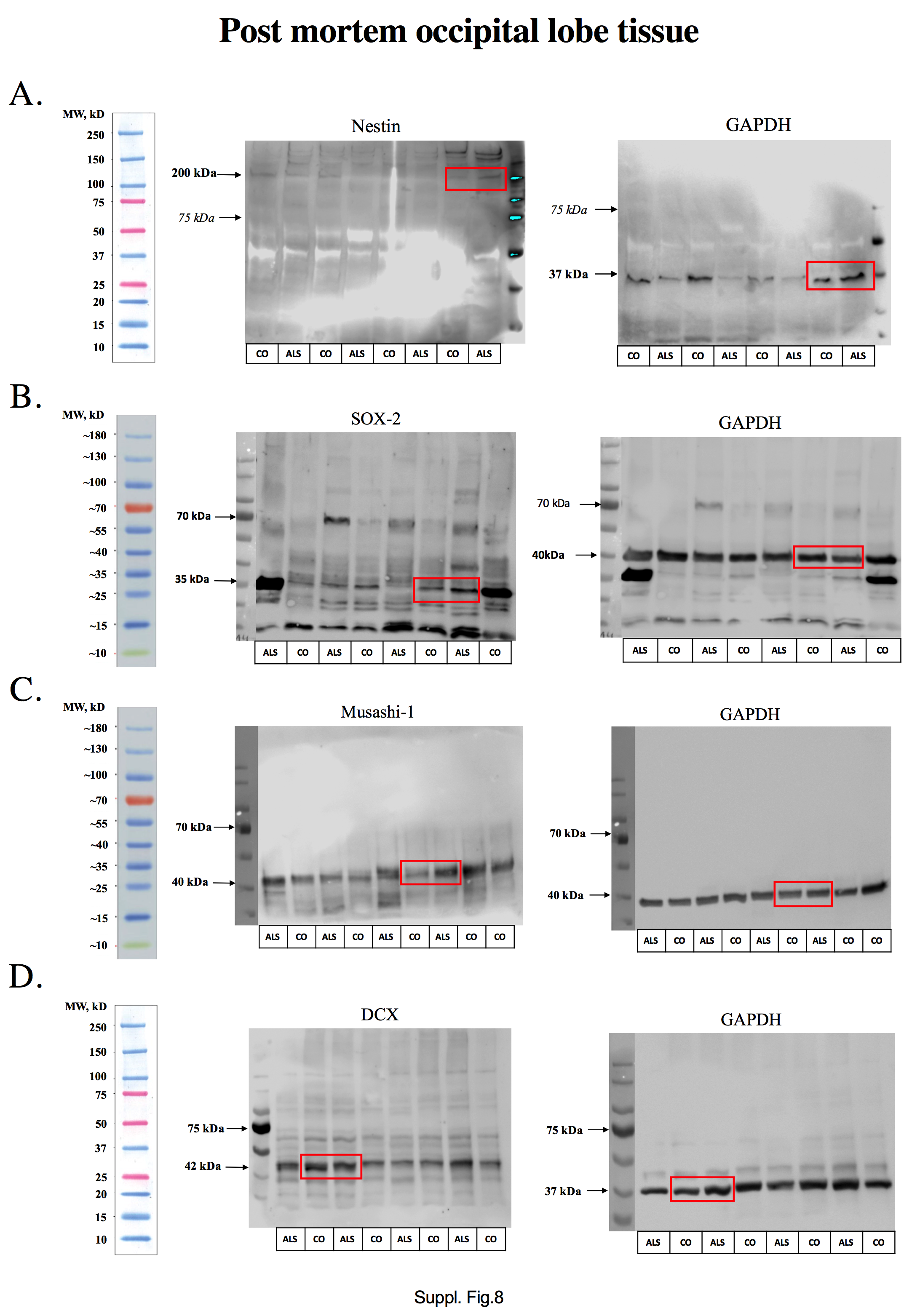

Supplement: Figure S8 — Whole Blots for Nestin (A), Sox-2 (B), Msi-1 (C), and DCX (D) within human postmortem occipital lobe tissue. Figures show protein standard ladder, whole blot for the respective analytes and the whole blot for the house-keeper GAPDH. Red squares indicate the representative blots shown within the main manuscript. [file Image_8.tiff]

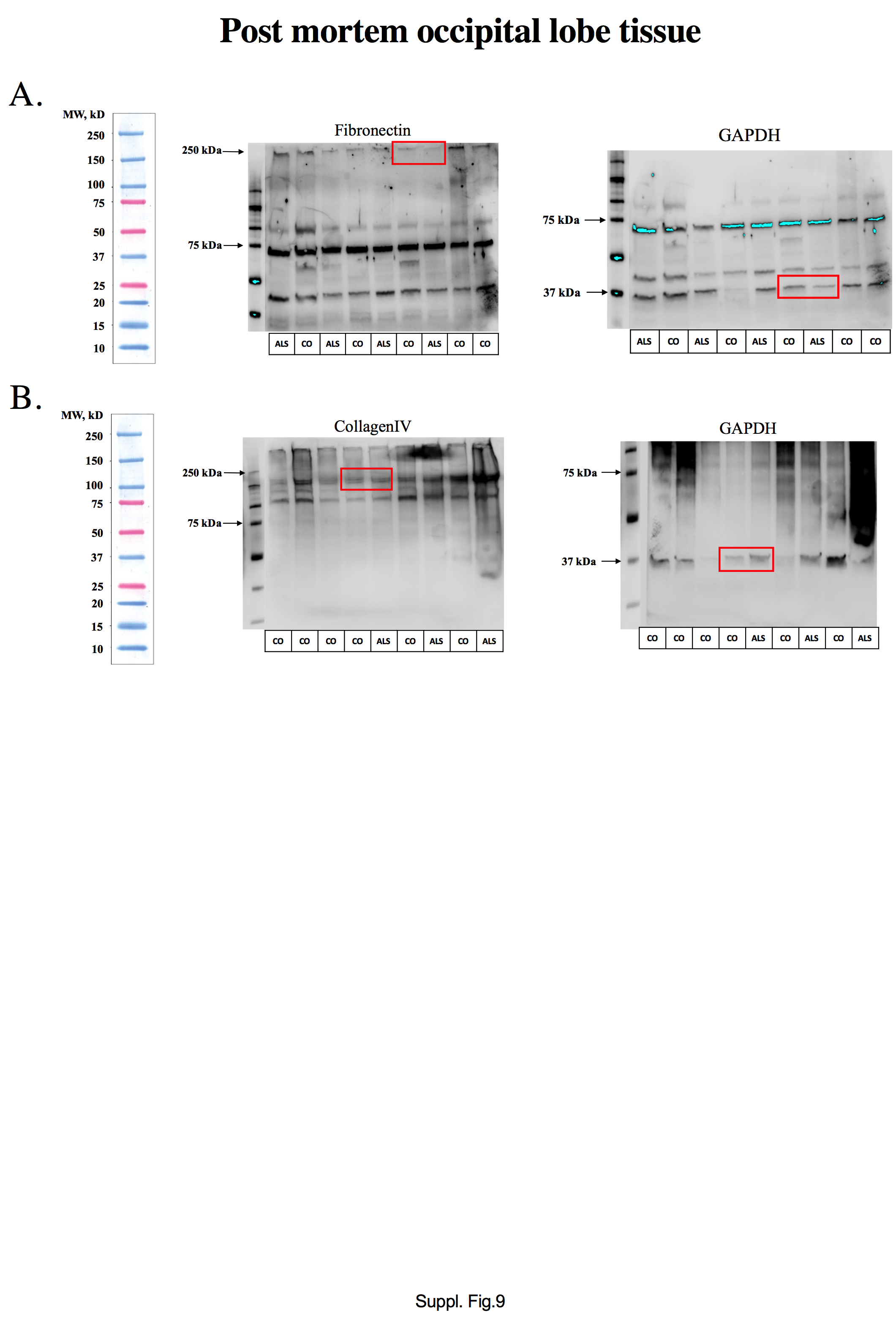

Supplement: Figure S9 — Whole Blots for fibronectin (A) and CollagenIV (B) within human postmortem occipital lobe tissue. Figures show protein standard ladder, whole blot for the respective analytes and the whole blot for the house-keeper GAPDH. Red squares indicate the representative blots shown within the main manuscript. [file Image_9.tiff]
