# Supplementary material for: The TGF-β System As a Potential Pathogenic Player in Disease Modulation of Amyotrophic Lateral Sclerosis
Source: Front Neurol. 2017 Dec 15;8:669. doi: 10.3389/fneur.2017.00669 (PMC5736544; doi:10.3389/fneur.2017.00669)
Supplement: Table S1 — Cytokines/chemokines analyzed in human serum samples of healthy controls and ALS patients but not shown as graphs within the manuscript. [file Table_1.PDF]

Analyte	Serum samples		p-value	Statistical test
IFNgamma	not significantly altered		0.203	Mann-Whitney test
IL-10	not significantly altered		0.6241	Mann-Whitney test
IL-12p70	not detectable		/	/
IL-13	not detectable		/	/
IL-1beta	not detectable		/	/
IL-2	not detectable		/	/
IL-4	not detectable		/	/
IL-6	not significantly altered		0.1261	Mann-Whitney test
IL-8	not significantly altered		0.8082	Mann-Whitney test
GM-CSF	not detectable		/	/
IL-12/IL-23p40	not significantly altered		0.6082	Mann-Whitney test
IL-16	not significantly altered		0.0948	Mann-Whitney test
IL-17A	not significantly altered		0.3214	Unpaired Student´s t-test
IL-1alpha	not detectable		/	/
IL-5	not detectable		/	/
Eotaxin	significantly altered	mean ± SEM Ctrl: 142 ± 16.27, n=11	0.0022	Unpaired Student´s t-test
		mean ± SEM ALS: 227.1 ± 13.39, n=37		
MDC	not significantly altered		0.2217	Unpaired Student´s t-test
Eotaxin-3	not significantly altered		0.137	Mann-Whitney test
MIP-1alpha	not detectable		/	/
IL-8(HA)	not detectable		/	/
FGF	not significantly altered		0.6082	Mann-Whitney test
Flt-1	not significantly altered		0.778	Unpaired Student´s t-test
VEGF-D	not significantly altered		0.9387	Mann-Whitney test
