# Supplementary material for: The TGF-β System As a Potential Pathogenic Player in Disease Modulation of Amyotrophic Lateral Sclerosis
Source: Front Neurol. 2017 Dec 15;8:669. doi: 10.3389/fneur.2017.00669 (PMC5736544; doi:10.3389/fneur.2017.00669)
Supplement: Table S2 — Cytokines/chemokines analyzed in human postmortem samples of healthy controls and ALS patients but not shown as graphs within the manuscript. [file Table_2.PDF]

Analyte	Spinal Cord		p-value	Statistical test	Motor Cortex	p-value	Statistical test	Occipital Lobe	p-value	Statistical test
IFNgamma	not significantly altered		0.1499	Mann-Whitney test	not significantly altered	0.896	Mann-Whitney test	not detectable	/	/
IL-12p70	not significantly altered		0.1251	Mann-Whitney test	not significantly altered	0.7438	Mann-Whitney test	not significantly altered	0.1031	Mann-Whitney test
IL-13	not significantly altered		0.2054	Mann-Whitney test	not significantly altered	0.7266	Unpaired Student´s t-test	not significantly altered	0.4719	Unpaired Student´s t-test
IL-2	not significantly altered		0.1499	Mann-Whitney test	not significantly altered	0.8388	Mann-Whitney test	not detectable	/	/
IL-6	not significantly altered		0.9459	Mann-Whitney test	not significantly altered	0.6471	Mann-Whitney test	not significantly altered	0.9273	Mann-Whitney test
IL-8	not significantly altered		0.3287	Mann-Whitney test	not significantly altered	0.3938	Mann-Whitney test	not significantly altered	0.1852	Mann-Whitney test
IL-12/IL-23p40	not significantly altered		0.5999	Mann-Whitney test	not detectable	/	/	not detectable	/	/
IL-16	significantly altered	median Ctrl: 231.8 , n = 14<br>median ALS: 380.1, n = 14	0.0058	Mann-Whitney test	not significantly altered	0.9479	Mann-Whitney test	not significantly altered	0.623	Mann-Whitney test
IL-17A	not significantly altered		0.8975	Unpaired Student´s t-test	not detectable	/	/	not detectable	/	/
IL-5	not significantly altered		0.2842	Unpaired Student´s t-test	not detectable	/	/	not detectable	/	/
MCP-4	not detectable		/	/	not detectable	/	/	not detectable	/	/
Eotaxin	not significantly altered		0.1335	Mann-Whitney test	not detectable	/	/	not detectable	/	/
Eotaxin-3	not significantly altered		0.3136	Mann-Whitney test	not detectable	/	/	not detectable	/	/
TARC	not significantly altered		0.2273	Mann-Whitney test	not detectable	/	/	not detectable	/	/
MIP-1alpha	not detectable		/	/	not detectable	/	/	not detectable	/	/
FGF	not significantly altered		0.9459	Mann-Whitney test	not detectable	/	/	not detectable	/	/
Flt-1	not significantly altered		0.069	Mann-Whitney test	not detectable	/	/	not detectable	/	/
VEGF-C	not detectable		/	/	not detectable	/	/	not detectable	/	/
VEGF-D	not detectable		/	/	not detectable	/	/	not detectable	/	/
